# The Cyclic AMP Receptor Protein Regulates Quorum Sensing and Global Gene Expression in Yersinia pestis during Planktonic Growth and Growth in Biofilms

**DOI:** 10.1128/mBio.02613-19

**Published:** 2019-11-19

**Authors:** Jeremy T. Ritzert, George Minasov, Ryan Embry, Matthew J. Schipma, Karla J. F. Satchell

**Affiliations:** a Department of Microbiology-Immunology, Northwestern University Feinberg School of Medicine, Chicago, Illinois, USA; b Center for Structural Genomics of Infectious Diseases, Feinberg School of Medicine, Northwestern University, Chicago, Illinois, USA; c Center for Genetic Medicine, Northwestern University, Chicago, Illinois, USA; UCLA School of Medicine

**Keywords:** *Yersinia pestis*, plague, Crp, RNA-seq, structure, quorum sensing, biofilms, catabolite repression, cyclic AMP, regulation of gene expression, three-dimensional structure

## Abstract

Cyclic AMP (cAMP) receptor protein (Crp) is an important transcriptional regulator of Yersinia pestis. Expression of *crp* increases during pneumonic plague as the pathogen depletes glucose and forms large biofilms within lungs. To better understand control of Y. pestis Crp, we determined a 1.8-Å crystal structure of the protein-cAMP complex. We found that compared to Escherichia coli Crp, C helix amino acid substitutions in Y. pestis Crp did not impact the cAMP dependency of Crp to bind DNA promoters. To investigate Y. pestis Crp-regulated genes during plague pneumonia, we performed RNA sequencing on both wild-type and Δ*crp* mutant bacteria growing in planktonic and biofilm states in minimal media with glucose or glycerol. Y. pestis Crp was found to dramatically alter expression of hundreds of genes in a manner dependent upon carbon source and growth state. Gel shift assays confirmed direct regulation of the *malT* and *ptsG* promoters, and Crp was then linked to Y. pestis growth on maltose as a sole carbon source. Iron regulation genes *ybtA* and *fyuA* were found to be indirectly regulated by Crp. A new connection between carbon source and quorum sensing was revealed as Crp was found to regulate production of acyl-homoserine lactones (AHLs) through direct and indirect regulation of genes for AHL synthetases and receptors. AHLs were subsequently identified in the lungs of Y. pestis-infected mice when *crp* expression was highest in Y. pestis biofilms. Thus, in addition to the well-studied *pla* gene, other Crp-regulated genes likely have important functions during plague infection.

## Abstract (precis)

Bacterial pathogens have evolved extensive signaling pathways to translate environmental signals into changes in gene expression. While Crp has long been appreciated for its role in regulating metabolism of carbon sources in many bacterial species, transcriptional profiling has revealed that this protein regulates many other aspects of bacterial physiology. The plague pathogen Y. pestis requires this global regulator to survive in blood, skin, and lungs. During disease progression, this organism adapts to changes within these niches. In addition to regulating genes for metabolism of nonglucose sugars, we found that Crp regulates genes for virulence, metal acquisition, and quorum sensing by direct or indirect mechanisms. Thus, this single transcriptional regulator, which responds to changes in available carbon sources, can regulate multiple critical behaviors for causing disease.

## INTRODUCTION

Yersinia pestis is the etiological agent of pneumonic, bubonic, and septicemic plague. While cases of plague are rare, Y. pestis evolved to adapt to environmental changes encountered during its life cycle in fleas, rodents, and mammals, including humans (1). The flea, buboes (lymph nodes), blood, and lungs differ in temperature, nutrients, and defense systems. To interpret changes in these environments, Y. pestis encodes more than two dozen two-component systems (2), three quorum sensing systems (3), and additional transcriptional regulators with specialized functions. This sensor network allows Y. pestis to translate changes in its extracellular environment into altered gene expression to promote growth and pathogenesis (4, 5).

The 3′,5′-cyclic AMP (cAMP) receptor protein (Crp) is required for Y. pestis virulence in mice (6). Crp is a 23.5-kDa transcriptional regulator that forms a dimer after binding its cAMP ligand (7). Dimerization allows activation or repression of gene expression by binding to the promoter region of its target genes (8). The activity of adenylate cyclase (CyaA) regulates intracellular concentrations of cAMP via the conversion of ATP into cAMP. This activity is increased at 37°C compared to lower temperatures, suggesting that cAMP-Crp signaling is important during mammalian infection (9, 10). In addition, phosphorylated EIIA from the phosphotransferase system activates CyaA when glucose is absent (11). During catabolite repression, the transport of glucose through PtsG depletes intracellular concentrations of phosphorylated EIIA as the phosphate group is transferred to the incoming glucose molecule.

While known for its role during catabolite repression and regulation of the *lac* operon, it is now appreciated that Crp regulates expression of other genes, including factors important during infection, to connect changes in glucose availability to regulation of bacterial behaviors. Across gammaproteobacteria, Crp regulates biofilm formation (12, 13), capsule production (14), the DNA damage response (15), toxin production (16, 17), luminescence (18), and iron acquisition (19).

Other proteins can regulate expression of the *crp* gene, suggesting that multiple input signals modulate expression of the Crp regulon. The two-component system PhoPQ and the small RNA (sRNA) chaperone Hfq regulate *crp* in Y. pestis (6, 20, 21). In turn, Crp regulates expression of the type III secretion system and the Pla protease essential for pneumonic plague (22–24). Crp also promotes biofilm formation via a mechanism involving the RNA-binding regulatory protein CsrA (13). Production of the main constituent of biofilms, poly-*N*-acetylglucosamine, is increased at 37°C in fully virulent strains of Y. pestis, suggesting additional roles for biofilm formation during mammalian infection (25). Further, we recently demonstrated increased expression of *crp* within biofilms in the lungs of Y. pestis-infected mice (26), revealing a link between *in vivo* biofilms, glucose availability, and essential virulence gene expression.

In this study, we utilized global transcriptional profiling of planktonic and biofilm states in the presence of glucose and glycerol to reveal previously unrecognized Crp-regulated genes in Y. pestis. We found that Crp indirectly represses genes required for siderophore biosynthesis and stimulates genes for carbohydrate uptake and metabolism, particularly for the use of maltose as an alternative carbon source. Unexpectedly, Crp was found to promote expression of the acyl-homoserine lactone (AHL) quorum sensing genes. Crp directly binds to the promoter of the AHL receptor, *ypeR*, and thereby controls efficient production of AHLs within biofilms.

## RESULTS AND DISCUSSION

### Experimental setup.

**FIG 1:**
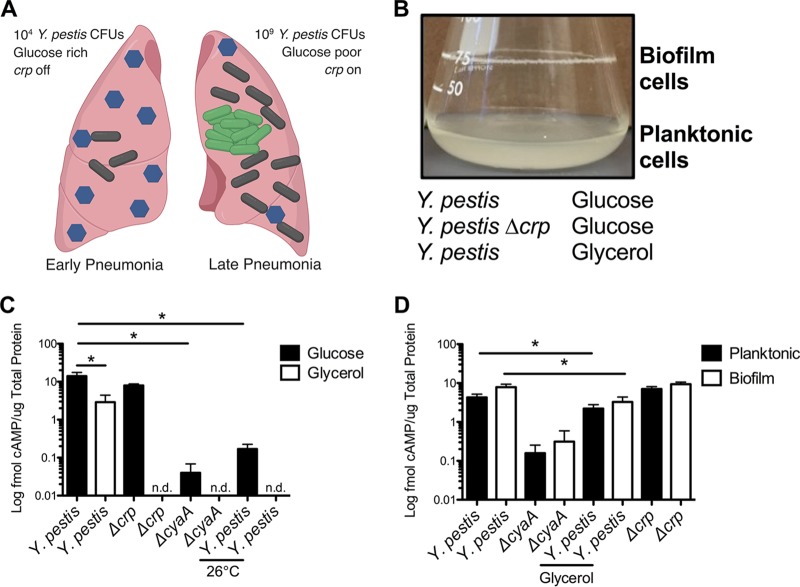
Experimental setup for RNA-seq. (A) Diagram of changes during progression of pneumonic plague. Blue hexagons represent glucose. Black and green bacilli represent wild-type Y. pestis and Y. pestis expressing *crp* in biofilms, respectively. (B) Culture (10 ml) of Y. pestis grown in TMH medium for 18 h at 37°C showing the biofilm formed at the air-liquid interface. RNA was isolated from wild-type Y. pestis or Y. pestis Δ*crp* cultures grown in TMH medium with 0.2% glucose or glycerol in triplicate under the described conditions. (C) Intracellular concentrations of cAMP in wild-type Y. pestis, Y. pestis Δ*crp*, or Y. pestis Δ*cyaA* grown for 6 h in TMH medium with 0.2% glucose (black bars) or glycerol (white bars) at 37°C or 26°C as indicated. n.d. = not done. (D) Intracellular concentrations of cAMP in Y. pestis, Y. pestis Δ*crp*, or Y. pestis Δ*cyaA* grown for 18 h in TMH medium with 0.2% glucose or glycerol in planktonic (black bars) or biofilm (white bars) states at 37°C. Data represent means and standard errors of the means (SEM) of results from three independent experiments. *, *P *< 0.05 from Student's *t* test.

Pneumonic plague is a biphasic disease consisting of an early noninflammatory phase and a damaging proinflammatory phase (27). As pneumonia develops, the lungs fill with Y. pestis, neutrophils, and fluid (Fig. 1A). Y. pestis proliferates to ∼10^9^ CFU, forms large biofilms in the lungs, and consumes all available glucose in the process. The declining concentration of glucose activates expression of *crp* within biofilms (26). Expression of the Crp-activated gene *pla*, which is required for Y. pestis to grow within the lungs and disseminate to other organs, also increases (22). To better understand the changes in the lung environment, we sought to identify additional Crp-regulated genes dependent upon growth in biofilms and under glucose-limiting conditions.

To recapitulate human infection conditions, Y. pestis was grown at 37°C with shaking in defined liquid culture media with glucose to mimic early pneumonia or with glycerol to mimic later infection after glucose is consumed. As the cultures were aerated by shaking, Y. pestis formed a biofilm at the air-liquid interface, thereby facilitating comparisons of levels of gene expression of the planktonic state representing early infection and the biofilm state of later infection (Fig. 1B). In addition, a Δ*crp* mutant was included in parallel to facilitate identification specifically of Crp-regulated genes under these conditions. The combined set of six experimental conditions allowed identification of glucose-, Crp-, and biofilm-dependent genes that may play a role during pneumonic plague infection.

### Carbon source and growth state do not affect cAMP requirement for Crp binding to DNA.

A potential concern with this experimental setup is that differences in expression of Crp-regulated genes could be affected by changes in the activity of CyaA and the phosphotransferase system (28), leading to differences in Crp activity rather than in expression of *crp*. To control for this possibility, we measured cAMP concentrations in Y. pestis under all experimental conditions. In contrast to the expected increase, we observed a decrease in cAMP concentrations in Y. pestis grown in glycerol (Fig. 1C). This was also reported in a study of Vibrio fischeri, in which intracellular cAMP levels were reduced but total concentrations of cAMP (including extracellular cAMP) were higher (29). Deletion of the *cyaA* adenylate cyclase gene reduced cAMP levels, while deletion of *crp* had no effect. cAMP concentrations were much higher in cells grown at 37°C versus 26°C, suggesting the importance of Crp regulation during mammalian infection. cAMP concentrations were not significantly affected in comparisons between plankton- and biofilm-grown cells (Fig. 1D). These data indicate that any changes in expression of Crp-regulated genes observed by RNA sequencing (RNA-seq) would not be due to differences in the cAMP levels.

**FIG 2:**
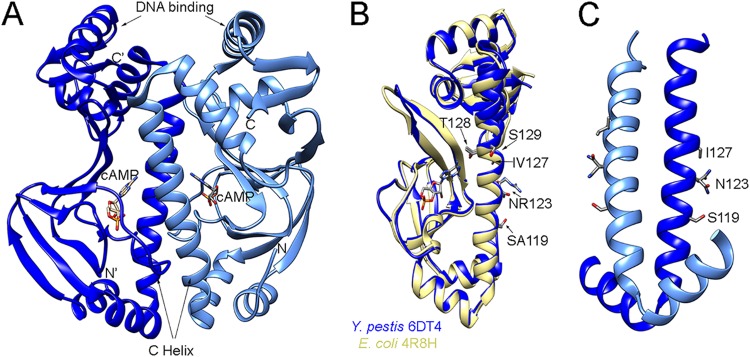
The structure of Y. pestis Crp closely aligns with that of E. coli Crp. (A) Structure (1.8-Å resolution) of Y. pestis Crp dimer bound to cAMP (PDB ID 6DT4). (B) Overlap of Y. pestis Crp chain A (blue) with E. coli Crp (yellow) (PDB ID 4R8H, chain A) (30). Residues that differ between Y. pestis and E. coli are marked, with the Y. pestis residue indicated first. Residues modified in E. coli that result in constitutive binding to DNA are also marked. (C) Dimer interface of Y. pestis Crp showing residues that differ from E. coli.

Another potential caveat concerning this experimental setup is the possibility that Crp could bind and regulate genes in Y. pestis entirely independently of cAMP. Earlier mutational studies on Escherichia coli showed that the specific double mutant variant T128L/S129I had extremely high cAMP-independent DNA binding affinity, comparable with the activity of cAMP-bound wild-type Crp (30). Y. pestis Crp shares 99% sequence homology with Crp from E. coli, with differences located on the C helix at positions 119, 123, and 127. We considered that the sequence differences might affect cAMP binding or dimerization and thus cause changes in DNA binding. To investigate this, we carried out structural studies using protein crystallography and solved the crystal structure of Y. pestis Crp in complex with cAMP. Crp was crystallized as a dimer, and each monomer had cAMP bound to the binding site (Fig. 2A). We compared the resulting structure to the structure of E. coli Crp bound to cAMP (PDB identifier [ID] 4R8H) (30). The structures aligned closely, with root mean square deviation (RMSD) values of 0.8 Å for 185 pruned Cα atom pairs and 1.1 Å for all 200 pairs (Fig. 2B). Notably, in the Y. pestis structure, C helix residues S119, N123, and I127 (unlike S129 in the constitutive binding mutant of E. coli Crp) are directed away from cAMP, suggesting that these altered amino acids should not impact cAMP binding (Fig. 2C). The side chain of residue N123 is involved in dimer formation and directly interacts with the side chain of E78 from another chain. The N123 exchange for arginine, which occurs in Y. pestis, disrupts this interaction, but the S119 replacement for alanine restores the hydrogen bond to E78; thus, these two mutations do not alter the stability of the dimer. V127 in Crp from E. coli is pointed away from dimerization interface, and it is not involved in the cAMP binding, so isoleucine at this position in Y. pestis does not have any impact that contributes to binding of cAMP or to dimer formation. As confirmation of our findings, an electrophoretic mobility shift assay (EMSA) performed in the presence of cAMP (see Fig. 5A) indicated that Crp requires cAMP to bind a DNA fragment that corresponds to the *pla* promoter. This cAMP dependence is also consistent with studies of Crp from other Y. pestis isolates and from Yersinia pseudotuberculosis (23, 31, 32).

Thus, cAMP concentration data, structural comparisons, and EMSA data suggest that Crp function is controlled by cAMP in Y. pestis but that the cAMP concentrations did not vary dramatically under the conditions surveyed in this study. Thus, we expect that variations in gene expression noted in this study would be linked specifically to Crp. This expectation is consistent with our prior finding that regulation of *pla* (between planktonic and biofilm-grown cells) depends on differential expression of Crp as opposed to changes in cAMP (26).

### Global analysis of gene expression.

**FIG 3:**
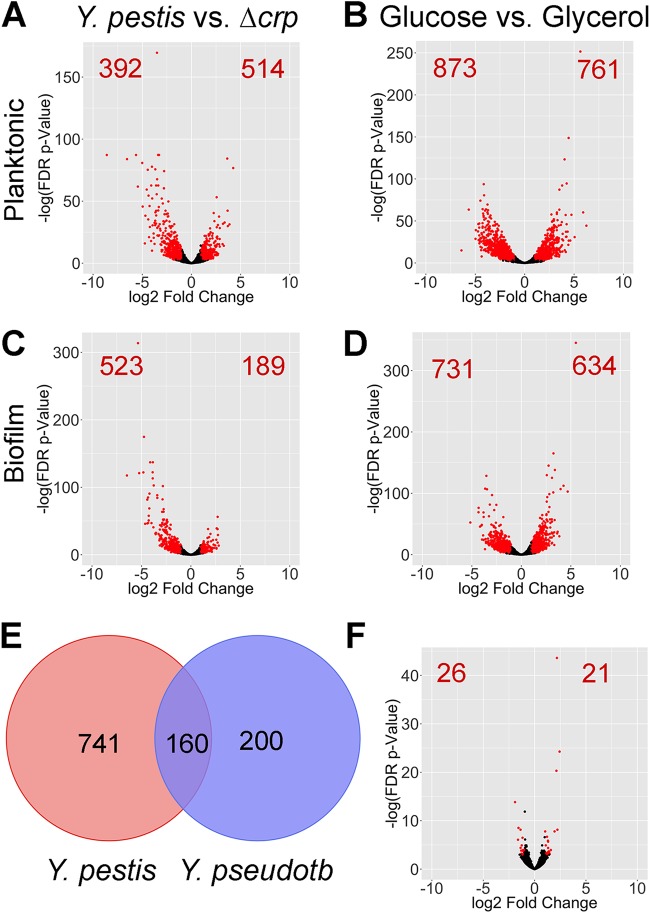
Identification of Crp-regulated, biofilm-regulated, and glucose-regulated genes in Y. pestis. (A to D) Volcano plots of genes differentially expressed between wild-type Y. pestis and Y. pestis Δ*crp* (A and C) and Y. pestis grown in 0.2% glucose versus glycerol (B and D) in the planktonic (A and B) and biofilm (C to D) states. Each individual dot represents a gene of Y. pestis; red dots and numbers indicate genes with log2 fold change greater than 1 or less than −1 and a false-discovery-rate *P* value of <0.05. (E) Venn diagram of Crp-regulated genes in Y. pestis from this study and Crp-regulated genes in the related Y. pseudotuberculosis (*Y. pseudotb*) YPIII strain (31). (F) Volcano plot of genes differentially expressed between Y. pestis grown in the planktonic and biofilm states in the presence of 0.2% glucose.

We subsequently performed RNA-seq on total RNA isolated from Y. pestis pCD1 and the Δ*crp* mutant under all experimental conditions. RNA from Y. pestis Δ*crp* growing planktonically and in biofilms was collected to identify Crp-regulated genes. Deep sequencing reads were mapped to the Y. pestis chromosome and plasmids pPCP1 and pMT1. Reads were also mapped to the location of annotated small RNAs (sRNAs) in Y. pestis (33). The raw RNA-seq data sets were deposited into GenBank (accession no. GSE135228). The cutoff value for significantly differentially expressed genes was set at log2 fold change greater than 1 or less than −1, with a false-discovery-rate (FDR) *P* value of <0.05. Complete lists of differentially expressed genes can be found in Data Sets S1 and S2 in the supplemental material. Principal-component analysis revealed close associations of most of the replicates across all conditions (see Fig. S1 in the supplemental material). Crp and carbon source altered expression of thousands of Y. pestis genes in planktonic and biofilm growth states (Fig. 3A to D). Indeed, 1,200 unique protein-coding genes (713 Crp-activated and 487 Crp-repressed genes) were impacted by the presence of *crp* whereas the presence of glucose altered expression of 1,872 unique genes between the planktonic and biofilm growth states.

10.1128/mBio.02613-19.1 FIG S1 Principal-component analysis of RNA-seq dataset. Data represent principal-component analysis of results of comparisons of the six different conditions and three different replicates. Download FIG S1, TIF file, 0.6 MB. Copyright © 2019 Ritzert et al. 2019 Ritzert et al. This content is distributed under the terms of the Creative Commons Attribution 4.0 International license.

Only 47 genes were differentially expressed strictly between plankton- and biofilm-grown cells (Fig. 3F). The low number of differentially expressed genes might be due to overlap of planktonic cells with biofilm cells at the air-liquid interface or to heterogeneous expression of genes throughout the biofilm. Genes required for biofilm formation such as those encoding diguanylate cyclases, e.g., *hmsT* and Y. pestis *0449* (*ypo0449*), were not differentially expressed between planktonic and biofilm cells (12, 34, 35). A similar observation in a study reported previously by Vadyvaloo et al. revealed that these genes were also not significantly different between planktonic cells and biofilms in flow cells (36). Genes previously known to influence biofilm formation, namely, *crp* (12), *csrA* (13), *rcsAB* (37), *rovM* and *rovA* (38), *phoPQ* (39), *fur* (40), and *hfq* (35), were also not differentially expressed. These data more likely suggest that genes for regulating biofilm formation are controlled by environmental factors, such as temperature and growth in the flea (41, 42). Carbon source is also known to play a role (13), and while we observed no difference in the levels of expression of the diguanylate cyclases in biofilms, they were significantly upregulated in glycerol compared to glucose (Data Set S1). An additional possibility is that the biofilms observed *in vitro* are the result of adhesion and autoaggregation of Y. pestis cells to each other and to the flasks due to the presence of the Ail outer adhesin. Expression of the *ail* gene was downregulated in Y. pestis biofilms in glucose and glycerol as shown by RNA-seq, but we cannot discount the possibility that sufficient Ail was still present to account for the biofilm or aggregation.

10.1128/mBio.02613-19.8 DATA SET S1 List of differentially expressed mRNAs in Y. pestis CO92 from comparisons between all test conditions. Download Data Set S1, XLSX file, 0.9 MB. Copyright © 2019 Ritzert et al. 2019 Ritzert et al. This content is distributed under the terms of the Creative Commons Attribution 4.0 International license.

Significant genes were categorized and enriched by biological process with Gene Ontology (GO) at GeneOntology.org by the use of Y. pestis as a reference list (Data Set S3) (43, 44). Categorizing Crp-activated genes in planktonic or biofilm cells returned results that demonstrated enrichment of DNA-templated transcriptional regulators involved in a wide range of biological processes, including catabolism of carbohydrates (*malT* and *araC*), amino acid metabolism (*leuO*), and quorum sensing (*ypeR* and *yspR*). This category was also enriched in glucose-repressed (i.e., glycerol-activated) genes with overlap of multiple transcriptional regulators, including *malT* and *ypeR.* We anticipated overlapping of Crp-activated and glucose-repressed genes, as well as overlapping of Crp-repressed and glucose-activated genes, on the basis of the function of cAMP-Crp. By filtering these data sets for genes increased in glycerol compared to glucose and increased in wild-type Y. pestis compared to Y. pestis Δ*crp*, we were able to better identify cAMP-Crp-activated (or repressed) genes (see Table S2 in the supplemental material). Importantly, the *pla* gene, known to be Crp-activated and glucose-repressed and active during the end stages of pneumonic plague (6, 23), filtered into the Crp-activated category. Several Crp-regulated genes from a previous microarray of Y. pestis (*pim*, *pst*, *ptsG*, *araF*, *rpoH*, *yfiA*, and *ompC*) (32) were also identified. While GO analysis identified quorum sensing transcriptional regulators *yspR* and *ypeR* as Crp activated, the corresponding acyl-homoserine lactone synthetase genes (*yspI* and *ypeI*) also were identified as CRP activated (Table S2).

10.1128/mBio.02613-19.5 TABLE S2 Crp-activated and -repressed genes. Download Table S2, DOCX file, 0.02 MB. Copyright © 2019 Ritzert et al. 2019 Ritzert et al. This content is distributed under the terms of the Creative Commons Attribution 4.0 International license.

10.1128/mBio.02613-19.9 DATA SET S2 List of differentially expressed sRNAs previously identified in Y. pestis CO92 from comparisons between all test conditions. Download Data Set S2, XLSX file, 0.2 MB. Copyright © 2019 Ritzert et al. 2019 Ritzert et al. This content is distributed under the terms of the Creative Commons Attribution 4.0 International license.

10.1128/mBio.02613-19.10 DATA SET S3 Gene ontology. Download Data Set S3, XLSX file, 0.03 MB. Copyright © 2019 Ritzert et al. 2019 Ritzert et al. This content is distributed under the terms of the Creative Commons Attribution 4.0 International license.

Furthermore, while we observed few differentially expressed genes in comparisons of Y. pestis in biofilms to Y. pestis in planktonic cells, genes and enriched pathways classified as activated or repressed by Crp or glucose differed between planktonic and biofilm cells. In other words, the determination of which genes or pathways are turned on by Crp or respond to changes in carbon source depends on whether Y. pestis is growing in a planktonic or biofilm state. We subsequently focused on identifying Crp-regulated genes that may play a role during the progression of pneumonic plague as Y. pestis forms biofilms and the environment switches from Crp repressive to Crp active.

We also observed overlapping of Crp-regulated genes identified here for Y. pestis and previously published expression profiling for Y. pseudotuberculosis compared to its isogenic Δ*crp* mutant (45). In total, 160 genes were shared across these data sets as differentially expressed in a manner dependent upon Crp, despite differing culture conditions and cutoffs for significance (Fig. 3E). An additional five genes (*rseC*, *rpsL*, *rpsG*, *rpmA*, and *ybiT*) were Crp activated in one species but Crp repressed in the other. Differences between the two data sets may also have resulted from the differing manners in which the *crp* gene is regulated among the two species. The small RNA chaperone Hfq is required for full production of Crp in Y. pestis but not in Y. pseudotuberculosis (6, 31). The PhoP response regulator is an activator of *crp* expression in one strain of Y. pestis (21), but variation in the DNA-binding domain of PhoP between *Yersinia* strains alters transcription of its target genes (46). In addition, these differences in regulation of the *crp* gene and in the extent to which genes are regulated by Crp likely result from changes in the promoter region of genes and reflect adaptations to the different environments that Y. pestis and Y. pseudotuberculosis inhabit.

### Crp indirectly represses expression of genes for yersiniabactin biosynthesis and uptake.

Among all the genes identified as controlled by Crp in the RNA-seq data set, it was noted that Crp represses expression of genes for metal acquisition (Data Set S1). These included genes for biosynthesis and uptake of the iron siderophore yersiniabactin (Ybt), such as *fyuA*, the gene for the Ybt receptor, and *ybtA*, the gene for the transcriptional regulator that controls expression of the Ybt locus (Table 1) (47). Consequently, the levels of expression of the *irp* genes, required for Ybt production, and genes for heme transport were also decreased, but the genes for the Yfe and Feo transport systems were not significantly differentially expressed (Table 1). Crp also represses expression of *ybtX* (*irp8* in strain CO92), a known virulence factor and trigger of inflammation during pneumonic plague (48, 49).

**TABLE 1:** Gene expression change due to Δ*crp* in metal acquisition genes

Group	Gene	Log2 fold change Y. pestis Δ*crp*^*a*^	
		Planktonic	Biofilm
Feo iron transport	*feoA*	+0.509	**+0.693**
	*feoB*	−0.120	−0.322

Iron regulation	*fur*	+0.590	+0.432

Ybt receptor	*fyuA*	**−2.552**	**−1.764**

Heme transport	*hmuR*	**−0.629**	−0.420
	*hmuS*	**−1.111**	−0.611
	*hmuT*	**−1.939**	**−0.925**
	*hmuU*	**−2.493**	**−1.067**
	*hmuV*	**−1.274**	−0.468

Ybt synthesis	*irp1*	**−2.575**	**−1.422**
	*irp2*	**−2.882**	**−1.880**
	*irp3*	**−2.434**	**−1.485**
	*irp4*	**−3.701**	**−2.187**
	*irp5*	**−3.297**	**−1.912**
	*irp6*	**−2.637**	**−2.706**
	*irp7*	**−3.168**	**−2.595**
	*irp8* (*ybtX*)	**−3.870**	**−2.587**

Ybt regulation	*ybtA*	−1.332	**−2.349**
	*ybtS*	**−1.398**	−0.590

Yfe iron transport	*yfeA*	+0.320	+0.552
	*yfeB*	−0.058	+0.097
	*yfeC*	−0.397	+0.191
	*yfeD*	+0.036	−0.179
	*yfeE*	+0.091	+0.162
	*yfeN*	+0.887	−0.222
	*yfeY*	+0.476	+0.252

Zinc transport	*znuA*	+0.053	+0.273
	*znuB*	**−1.345**	**−1.249**
	*znuC*	−0.741	−0.425

a A positive number indicates that the gene is more highly expressed in the Y. pestis Δ*crp* mutant and thus is Crp activated. A negative number indicates that the gene is Crp repressed. Bold data represent FDR *P* of <0.05.

**FIG 4:**
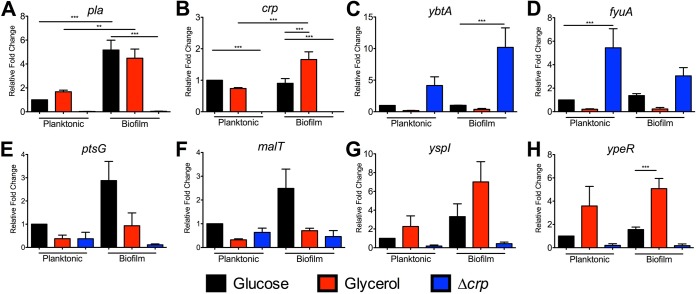
qRT-PCR of Crp-regulated genes identified from RNA-seq. RNA was isolated from cultures grown under the same conditions for RNA-seq. qRT-PCR for (A) *pla*, (B) *crp*, (C) *ybtA*, (D) *fyuA*, (E) *ptsG*, (F) *malT*, (G) *yspI*, and (H) *ypeR*. Data represent the means and SEM of results from four independent experiments. *, *P *< 0.05; **, *P *< 0.01; ***, *P *< 0.001 (from one-way ANOVA with Bonferroni’s multiple-comparison test).

**FIG 5:**
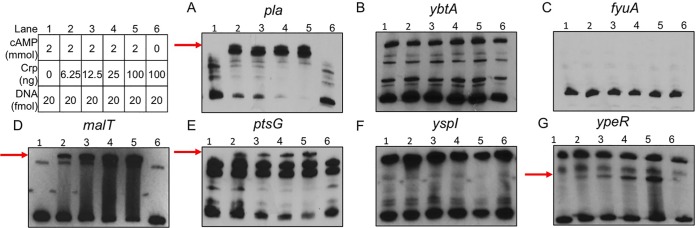
Crp binds to *pla*, *malT*, *ptsG*, and *ypeR* promoter sequences. EMSAs using purified Crp protein incubated with DNA fragments corresponding to promoters for (A) *pla*, (B) *ybtA*, (C) *fyuA*, (D) *malT*, (E) *ptsG*, (F) *yspI*, and (G) *ypeR.* Red arrows denote shifted band present in lanes containing Crp protein and cAMP. Table denotes concentrations of Crp protein, cAMP, and promoter DNA used in each reaction.

Reverse transcription-quantitative PCR (qRT-PCR) results supported the RNA-seq results, as transcript levels of *ybtA* and *fyuA* were reduced in glycerol-grown cultures and were also increased in the Δ*crp* mutant, albeit not to a statistically significant degree (Fig. 4C and D). Crp repression was observed in both the biofilm and planktonic states. EMSAs were also performed to determine whether Crp directly binds to the promoters of these genes. In contrast to Crp binding to the DNA sequence corresponding to the *pla* promoter in a cAMP-dependent manner (Fig. 5A), Crp did not bind to sequences corresponding to the *ybtA* or *fyuA* promoters (Fig. 5B and C). Thus, the control of Ybt by Crp is likely indirect. Crp did not alter expression of *fur* or of the RyhB sRNAs, known regulators of iron acquisition (Data Sets S1 and S2).

It is surprising to find that *ybt* genes are Crp repressed whereas other virulence factors, such as *pla* and *psa*, are Crp activated. Iron acquisition is critical for Y. pestis pathogenesis in multiple infectious routes (50) and would have been predicted to be required in the potential iron-limiting environment of the lung. It is possible that Ybt is necessary early in pneumonia and expressed when glucose is plentiful and *crp* expression is low. As the pneumonia progresses to the proinflammatory phase and host cells die of pyroptosis (51), iron is acquired by other means or is more available and Crp turns off expression of the Ybt genes indirectly.

### Crp is required for growth on nonglucose sugars and directly binds to the *malT* promoter.

Another potential important set of genes controlled by Crp during infection consists of those essential for the use of alternative carbon sources as glucose is depleted in the lung. Many of the Crp-activated genes identified by RNA-seq and enriched pathways identified by GO analysis are involved in metabolism or transport (Data Sets S1 and S3). This is not surprising given the historical role of Crp in regulating the *lac* operon and genes for alternative sugar metabolism mechanisms (52). In our RNA-seq data, we found that Crp activated expression of *ptsG*, which is a gene that is required for acquiring glucose during pneumonia and that is also important during bubonic plague (26). Crp was also found to increase expression of *malT*, the transcriptional activator of maltose metabolism (53). Genes *ptsG* and *malT* were confirmed to be controlled by Crp, as transcript levels were lower in the Δ*crp* mutant than in wild-type Y. pestis as shown by qRT-PCR (Fig. 4E and F), but transcript levels were not increased when grown in glycerol. This regulation was direct, as EMSAs demonstrated that Crp directly bound to the promoter sequences for *ptsG* and *malT* (Fig. 5D and E). In addition, the binding of Crp to these promoters required cAMP.

**FIG 6:**
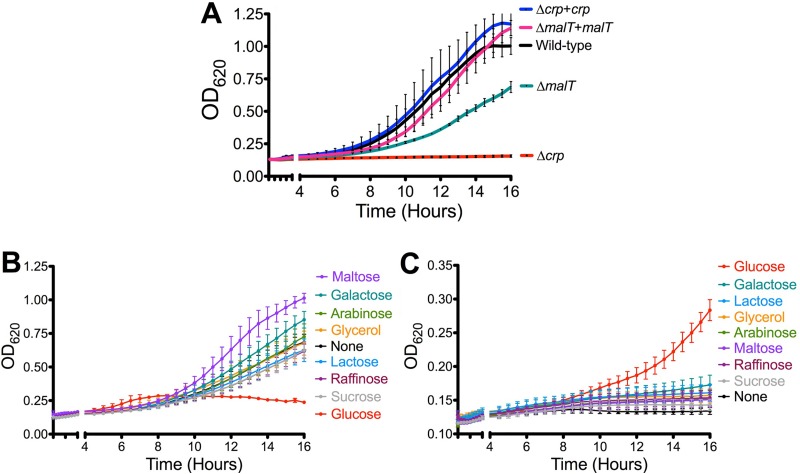
Crp is required for growth on nonglucose sugars. (A) Growth of wild-type Y. pestis and denoted strains on TMH medium plus 0.2% maltose for 16 h at 37°C. (B and C) Growth of wild-type Y. pestis (B) and Y. pestis Δ*crp* (C) in TMH medium supplemented with no carbon source or 0.2% of the indicated carbon sources for 16 h at 37°C. Data are combined from three independent experiments, and error bars represent means and SEM.

The direct regulation of the *malT* promoter by Crp suggested that maltose could be an important alternative to glucose to support growth of Y. pestis. Indeed, while Y. pestis grew well in thoroughly modified Higuchi (TMH) medium with maltose (Fig. 6A), the Δ*crp* mutant did not grow with maltose as the sole carbon source. Deletion of *malT* also reduced growth, although this mutant grew better than Y. pestis Δ*crp*, suggesting that Crp activates expression of additional genes involved in maltose metabolism. Complementation of *crp* and *malT* restored growth of the Δ*crp* and Δ*malT* mutants, respectively. In addition to the results seen with maltose, Y. pestis grew better in TMH medium supplemented with galactose than in TMH medium alone (Fig. 6B). Genes for galactose catabolism were not significantly different between wild-type Y. pestis and Y. pestis Δ*crp*, but genes in the *araF* operon for arabinose catabolism were found to be dependent on *crp* for expression (Data Set S1). In TMH medium supplemented with glucose, Y. pestis formed a biofilm at the air-liquid interface that resulted in lowered observed growth in this assay (Fig. S2A). Such robust biofilm formation was not observed in the presence of the other carbon sources. In contrast, Y. pestis Δ*crp* grew only in TMH medium with glucose (Fig. 6C), suggesting that Crp-activated genes are necessary for metabolism of nonglucose carbon sources. However, which carbon sources Y. pestis uses in the lungs after glucose is consumed is unknown.

10.1128/mBio.02613-19.2 FIG S2 Biofilm formation after growth in TMH medium. The images show results of crystal violet staining of (A) Y. pestis and (B) Y. pestis Δ*crp* biofilms formed after 16 h of growth in TMH medium supplemented with indicated carbon sources. Wells were stained as described previously (35). Download FIG S2, TIF file, 0.3 MB. Copyright © 2019 Ritzert et al. 2019 Ritzert et al. This content is distributed under the terms of the Creative Commons Attribution 4.0 International license.

### Crp directly and indirectly regulates expression of quorum sensing genes.

**FIG 7:**
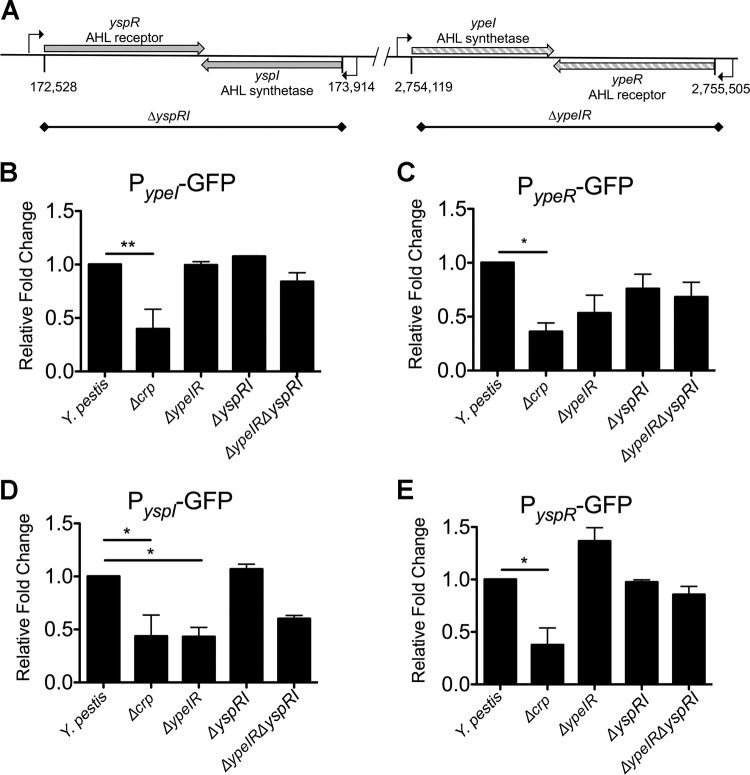
Crp directly and indirectly regulates expression of Y. pestis quorum sensing. (A) Schematic of arrangement of *yspIR* and *ypeRI* loci in genome of Y. pestis CO92. (B to E) Results of GFP reporter assays performed using the (B) P*_ypeI_*-GFP, (C) P*_ypeR_*-GFP, (D) P*_yspI_*-GFP, and (E) P*_yspR_*-GFP reporters integrated into wild-type Y. pestis and the Δ*crp*, Δ*ypeIR*, Δ*yspIR*, and Δ*ypeIR* Δ*yspIR* mutant strains. Background fluorescence was subtracted, and the remaining signal was normalized to wild-type Y. pestis, the value for which was set to 1. Data represent the means and SEM of results from three independent experiments. *, *P *< 0.05; **, *P *< 0.01 (from one-way ANOVA with Bonferroni’s multiple-comparison test).

A final class of potentially important Crp-regulated genes identified in this study consists of those associated with the LuxIR-based acyl-homoserine lactone (AHL) quorum sensing systems. The gene pairs in the Y. pestis genome are 100% identical to the AHL synthetase genes (*yspI* and *ypeI*) convergently transcribed with the receptors (*yspR* and *ypeR*) (Fig. 7A). Crp positively controlled expression of both the AHL synthetase genes and the AHL receptors (Table 2). qRT-PCR demonstrated that expression of *yspI* and *ypeR* in glycerol was increased in wild-type Y. pestis compared to Y. pestis Δ*crp*, similarly to the positive-control *pla* (Fig. 4A and G to H; not statistically significant). Moreover, expression of the AHL synthetase *yspI* gene was further increased during biofilm growth. A cAMP-Crp dependent shift of the DNA sequence corresponding to the *ypeR* promoter was observed by EMSA (Fig. 5E). However, Crp did not bind to *yspI* promoter sequence (Fig. 5F), suggesting that regulation of this gene is indirect.

**TABLE 2:** Gene expression change due to Δ*crp* in quorum sensing genes

Group	Gene	Log2 fold change Y. pestis Δ*crp*^*a*^	
		Planktonic	Biofilm
AHL	*ypeI*	**+1.817**	**+1.555**
	*ypeR*	**+1.624**	**+1.446**
	*yspI*	**+2.792**	**+2.709**
	*yspR*	**+2.731**	**+2.189**

AI-2	*luxS*	−0.665	**−1.099**

a A positive number indicates that the gene is Crp activated. A negative number indicates that the gene is Crp repressed. Bold data represent FDR *P* of <0.05.

In Y. pseudotuberculosis, expression of the same quorum sensing genes is autoregulated and regulated by each other and and Crp activated (31, 54). Both sets of synthetases and receptors are expressed from separate promoters antiparallel to each other (Fig. 7A). To determine if a similar pattern occurs in Y. pestis, green fluorescent protein (GFP) reporters containing the promoter regions of *yspR*, *yspI*, *ypeI*, and *ypeR* were integrated into wild-type Y. pestis, Y. pestis Δ*crp*, Y. pestis with deletion of *ypeI* plus *ypeR* (Δ*ypeIR*), Y. pestis with deletion of *yspR* plus *yspI* (Δ*yspRI*), or Y. pestis with deletion of all four AHL genes (Δ*yspRI* Δ*ypeIR*). Expression of all four reporters was reduced approximately 50% in Y. pestis Δ*crp*, confirming RNA-seq and qRT-PCR data showing that Crp stimulates expression of the *ype* and *ysp* AHL genes (Fig. 7B to E). Expression of the P*_ypeI_-*GFP reporter showed no dependence upon *ysp* or *ype* genes. The P*_ypeR_-*GFP reporter expression was reduced in the Δ*ypeIR* and Δ*yspRI* Δ*ypeIR* mutants, suggesting that *ypeR* expression may be positively autoregulated, but the differences were not statistically significant. Exactly the opposite occurs in Y. pseudotuberculosis, wherein the *ypeIR* (*ypsIR*) genes repress their own expression at 37°C (54). Expression of the P*_yspI_-*GFP reporter was reduced 2-fold in Y. pestis Δ*ypeIR*, suggesting that the reduction in *yspI* expression in Y. pestis Δ*crp* could have been due in part to reduced expression of *ypeR* (Fig. 4G). Similarly, the *yspI* homolog in Y. pseudotuberculosis is dependent on *ypeR* (*ypsR* in Y. pseudotuberculosis) (54) and expression of *ypeIR* and *yspIR* homologs in Y. pseudotuberculosis YPIII may also be Crp regulated (31).

Taken together, these data suggest direct regulation by Crp at the *ypeR* promoter and indirect regulation of *yspI* through *ypeR* resulting in stimulation of genes in glucose-limited media in biofilm. Despite the coding sequences of these genes being 100% identical, the quorum sensing genes are regulated differently between and within Y. pestis and Y. pseudotuberculosis.

### Crp regulates production of AHLs *in vitro*.

**FIG 8:**
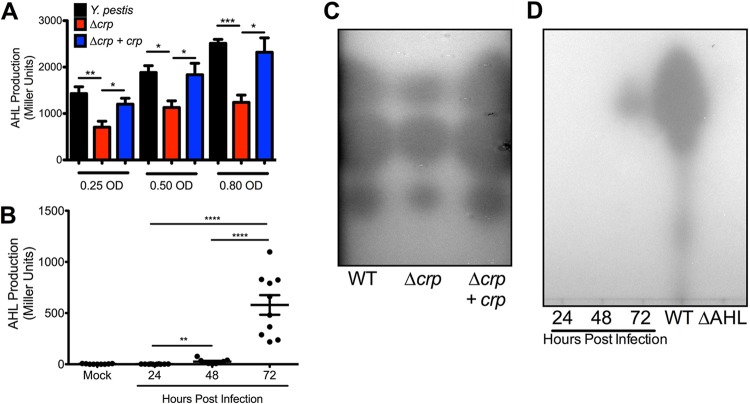
AHL production requires Crp and increases during pneumonic plague. (A) Comparison of levels of AHL production by wild-type Y. pestis and the Δ*crp* and Δ*crp+crp* strains at equivalent cell densities. (B) β-Galactosidase activity from AHL reporter strain of R. radiobacter incubated with BALF collected from mock-infected mice or mice infected intranasally with Y. pestis for 24, 48, or 72 h. (C) Representative TLC plate of AHL extracts collected from wild-type (WT) Y. pestis and the Y. pestis Δ*crp* and Δ*crp+crp* strains grown to an OD_620_ of 0.80 (wild-type Y. pestis and Y. pestis Δ*crp+crp* after 6 h, Y. pestis Δ*crp* after 12 h). From top to bottom, the proportions of the intensity of the AHL spots from Y. pestis Δ*crp* were 77%, 83%, and 76% those of the area of the wild-type strain corresponding to *N*-(3-oxohexanoyl)-l-homoserine, *N*-hexanoyl-l-homoserine, and *N*-octanoyl-l-homoserine lactones, respectively (55). Data are representative of results from three-independent experiments. Spot intensities were calculated in Fiji. (D) Representative TLC plate of AHL extracts collected from BALF samples. *, *P *< 0.05; **, *P *< 0.01; ***, *P *< 0.001; ****, *P *< 0.0001 (from Student's *t* test).

Given the decrease in expression of *yspI* and *ypeI* in Y. pestis Δ*crp*, production of AHLs should also have been reduced. Cell-free supernatants from Y. pestis strains were collected during planktonic growth *in vitro* and were used to stimulate the Rhizobium radiobacter AHL bioreporter. Levels of AHLs produced by Y. pestis increased with cell density during exponential-phase growth as expected for quorum sensing (Fig. S3A). In contrast, AHL production in Y. pestis Δ*crp* increased only slightly despite a 6-fold increase in bacterial density. Because the growth rate of Y. pestis Δ*crp* was found to be reduced, AHL concentrations were also compared between strains at similar cell densities. Even accounting for cell density, AHL production was reduced in the Δ*crp* strain (Fig. 8A). These data suggest that the reduction in AHL production was due to reduced *ypeI* and *yspI* expression and not to differences in cell density or growth of Y. pestis Δ*crp*. To determine if Crp alters production of specific AHLs, extracts were developed by thin-layer chromatography (TLC). Three spots were observed in all three strains that corresponded to 3-oxo-C6-homoserine lactone (3-oxo-C6-HSL), C6-HSL, and C8-HSL (Fig. 8C, top to bottom) as reported previously for Y. pestis (55). However, the areas of each of the spots from Y. pestis Δ*crp* were smaller. No spots were detectable in extracts collected from the Δ*ypeIR* Δ*yspRI* mutant strain (ΔAHL, Fig. 8D).

10.1128/mBio.02613-19.3 FIG S3 Crp does not regulate AI-2-based quorum sensing. (A) Growth (OD_620_, left *y* axis, solid lines) and AHL production (Miller units, right *y* axis, dotted lines) of wild-type (black), Δ*crp* (red), Δ*crp+crp* (blue), and Δ*ypeIR* Δ*yspIR* (purple) Y. pestis strains. Strains were grown in TMH medium with 0.2% glucose at 37°C. The OD_620_ of cultures was measured every 2 h, and culture supernatants were collected and used to stimulate a R. radiobacter bioreporter. (B) AI-2 production measured using the V. harveyi MM32 bioreporter from supernatants collected as described for panel A. (C) Comparison of AI-2 production by wild-type, Δ*crp*, and Δ*crp+crp*Y. pestis strains at equivalent cell densities. Data represent the means and SEM of results from four independent experiments. (D) Luminescence production from the AI-2 reporter strain of V. harveyi incubated with BALF collected from mock-infected mice or mice infected intranasally with Y. pestis for 24, 48, or 72 h. Data are combined from two independent animal infections with *n* = 5 at each time point. *, *P *< 0.05; **, *P *< 0.01; ***, *P *< 0.001; ****, *P *< 0.0001 (from Student’s *t* test). Download FIG S3, TIF file, 1.0 MB. Copyright © 2019 Ritzert et al. 2019 Ritzert et al. This content is distributed under the terms of the Creative Commons Attribution 4.0 International license.

To determine whether AHL molecules are produced during lung infection, bronchoalveolar lavage fluid (BALF) was collected from mice infected with fully virulent Y. pestis. AHLs were undetectable in BALF from mock-infected mice and mice at 24 h postinfection (hpi) (Fig. 8B). Only low levels of AHLs were detectable in BALF at 48 hpi, while BALF contained a 10-fold increase in AHLs at 72 hpi. Similar trends were present for AHLs from BALF developed by TLC plates (Fig. 8D). The significant increase in AHLs at 72 hpi correlates with the time frame during pneumonia when Y. pestis depletes glucose from the lungs, forms large biofilms, and, as a result, expresses *crp* at a high level (Fig. 1A) (26). The formation of biofilms in the lungs may provide a favorable environment for amplification of quorum sensing-based gene regulation. Indeed, *yspI* was more highly expressed *in vitro* in glycerol-grown biofilms than in glucose-grown biofilms (Fig. 4G). Taken together, these data provide strong evidence for Crp-dependent production of AHLs and induction of quorum sensing during lung infection. Transcriptome studies have linked Y. pestis quorum sensing mutants to defects in multiple behaviors (75). Deletion of both synthetase-receptor pairs impairs the ability of Y. pestis to make biofilms and metabolize maltose (55, 56). Both of these phenotypes are also Crp dependent (Fig. 6) (12) and suggest that connecting the Crp regulon to the quorum sensing regulon may allow Y. pestis to cooperatively regulate gene expression under conditions in which glucose is depleted and bacterial density is high.

While multiple measurements indicated that Crp regulates AHL-based quorum sensing, autoinducer-2 (AI-2)-based or LuxS-based quorum sensing is not Crp regulated in the planktonic state (Table 2; see also Data Set S1). The levels of AI-2 secretion were not significantly different between wild-type Y. pestis and Y. pestis Δ*crp* at equivalent cell densities in planktonic culture (Fig. S3B and C). In contrast to AHLs, the concentration of AI-2 in BALF samples increased in a stepwise manner from 24 to 48 to 72 hpi as Y. pestis density grew within the lungs even prior to the period of increased expression of *crp* (Fig. S3D) (26). These data suggest that AI-2 production is not dependent on Crp but rather that bacterial cell density increases between 24 and 72 hpi.

### Conclusions.

**FIG 9:**
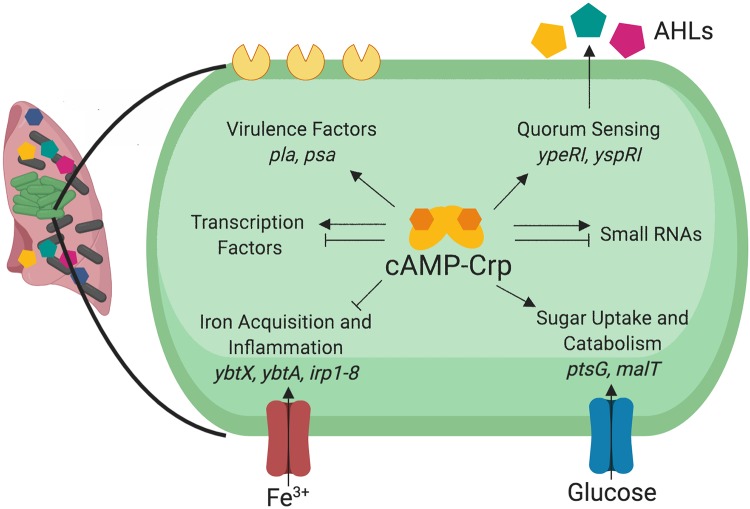
Model of identified Crp-activated genes and pathways in Y. pestis. Crp requires cAMP in order to regulate multiple behaviors of Y. pestis, including virulence, quorum sensing, iron acquisition, inflammation, and sugar uptake and catabolism. cAMP-Crp also regulates expression of hundreds of other mRNAs, including transcription factors, and small noncoding RNAs.

In this study, we solved the crystal structure of Y. pestis Crp while determining its regulon under planktonic and biofilm conditions. While *crp* is structurally similar to the E. coli Crp gene, the modes of regulation of the *crp* gene differ among pathogenic *Yersinia* spp., which colonize distinct environmental niches. Overall, transcription profiling conducted in this study revealed comprehensive insight into how Y. pestis adapts to growth in the biofilm state and the role of Crp and carbon sources during this growth. This is specifically relevant in the lungs, where Crp, through sensing of depletion of glucose, may serve as a switch turning on or off multiple behaviors of Y. pestis as disease progresses (26) (Fig. 9).

A unique result revealed by our transcriptional profiling was the observed indirect repression of Ybt genes by Crp (Table 1; see also Table S2). The Ybt and *irp* genes are critical for iron acquisition, virulence, and inflammation during pneumonic plague (48–50). Expression of these genes may be higher during the first 48 hpi, when expression and activity of Crp are low (Fig. 1A). After 48 hpi, Crp activates a second set of virulence factors such as *pla* and *psa* as disease progresses, correlating with the biphasic nature of pneumonic plague (23, 27, 32). The regulation of iron acquisition genes is unique to Y. pestis compared to Y. pseudotuberculosis. Even though these closely related species share 100% identical Crp proteins, the expression of the *crp* gene and the environments inhabited by these species may afford different sets of regulated genes.

We have expanded the pool of directly Crp-activated genes in Y. pestis to include genes for quorum sensing and alternative metabolisms. As glucose becomes a limiting resource, genes for carbohydrate catabolism and uptake are activated to support growth of Y. pestis. Within biofilms in the lungs, AHL-dependent quorum sensing is likely amplified due to the close proximity of cells. Here, we report production of AHLs in the lungs of plague-infected mice correlating with the time during which expression of *crp* is highest and Y. pestis is growing within biofilms of the lungs. Thus, Crp is directly regulating not only its own genes but also other transcriptional regulators and regulons (Fig. 9). These additional pathways may direct additional *Yersinia* behaviors and may serve as a link to the examples of indirect regulation observed in our data set. Future experimentation performed on the differentially expressed mRNAs and sRNAs identified in this study will provide additional information on biofilm-regulated and Crp-regulated genes and behaviors in Y. pestis and on their importance during pneumonia.

## MATERIALS AND METHODS

### Bacterial strains, media, and growth conditions.

The bacterial strains, plasmids, and primers used in this study are listed in Tables S3 and S4 in the supplemental material. Y. pestis strains (6, 74) were passaged on Difco brain heart infusion (BHI) agar (Becton, Dickinson) and into 2 ml of BHI broth or of the defined media, TMH (57), supplemented with 0.2% glucose for overnight growth at 26°C. Unless otherwise stated, overnight cultures in TMH medium were subcultured at an optical density at 620 nm (OD_620_) of 0.1 into 10 ml of TMH medium with 0.2% glucose in 125-ml Erlenmeyer flasks incubated at 37°C with shaking at 250 rpm for measuring planktonic and biofilm cell differences. Planktonic cells were removed from the flask with a serological pipette. Biofilms were scraped and resuspended in phosphate-buffered saline (PBS) warmed to 37°C. For growth assays, overnight cultures were subcultured at an OD_620_ of 0.1 into 200 μl of fresh TMH medium with 0.2% of indicated carbon sources at 37°C in a 96-well plate. Absorbance was measured with a Molecular Devices Spectramax M5 microplate reader. E. coli strains were passaged on Luria-Bertani (LB) agar or in LB broth at 37°C. Vibrio harveyi MM32 (75) was cultured in ATCC 2034 medium or LB with kanamycin. R. radiobacter was passaged on BHI medium with gentamicin. Ampicillin (100 μg/ml), kanamycin (50 μg/ml), and gentamicin (15 μg/ml) were added as necessary.

10.1128/mBio.02613-19.6 TABLE S3 List of bacterial strains and plasmids used in this study. Download Table S3, DOCX file, 0.02 MB. Copyright © 2019 Ritzert et al. 2019 Ritzert et al. This content is distributed under the terms of the Creative Commons Attribution 4.0 International license.

10.1128/mBio.02613-19.7 TABLE S4 List of oligonucleotides used in this study. Download Table S4, DOCX file, 0.02 MB. Copyright © 2019 Ritzert et al. 2019 Ritzert et al. This content is distributed under the terms of the Creative Commons Attribution 4.0 International license.

### Construction of new strains and plasmids.

Y. pestis mutants containing deletions in *crp*, *ypeIR*, *yspRI*, and *luxS* were generated by lambda red recombination (6). The Δ*ypeIR* Δ*yspRI* double mutant was generated by deletion of the *ypeIR* locus from the Δ*yspRI* mutant. Y. pestis Δ*crp* and Δ*malT* were complemented in *trans* by cloning the *crp* or *malT* codons plus 500 bp flanking the genes into pUC18-R6k-miniTn7t by Gibson assembly. The resulting plasmid was integrated into the Tn*7* site (58). GFP reporters were also integrated into the Tn*7* site. Amplification of 500 bp upstream of the promoters for *yspI*, *yspR*, *ypeI*, and *ypeR* was performed by PCR, and the amplification products were adjoined to the codons of *gfp* by overlap extension PCR as described previously (35).

### RNA extraction, sequencing, and qRT-PCR.

Total RNA was isolated from Y. pestis cultures as described previously (59) except that RNA protect reagent (Qiagen) was added to planktonic or biofilm cells in PBS. RNA was treated with 1 μl of Turbo DNase (Invitrogen Life Technologies) for 30 min at 37°C followed by addition of another 1 μl for 30 min following the manufacturer’s instructions.

Next-generation RNA sequencing was carried out at the Northwestern University NUSeq Core Facility. Total RNA was checked for quality and quantity on an Agilent Bioanalyzer 2100 and a Qubit fluorometer. An Illumina TruSeq stranded total RNA library preparation kit was used to prepare sequencing libraries from 500 ng of total RNA samples according to the manufacturer’s instructions without modifications. This procedure includes depletion of rRNA by the use of a Ribo-Zero rRNA removal kit (bacteria), purification and fragmentation of the remaining RNA, cDNA synthesis, 3′-end adenylation, Illumina adapter ligation, and library PCR amplification and validation. An Illumina NextSeq 500 sequencer was used to sequence the libraries with the production of single-ended, 75-bp reads.

The quality of DNA reads, in fastq format, was evaluated using FastQC. Adapters were trimmed, and reads that were of poor quality or that aligned to rRNA sequences were filtered out. The cleaned reads were aligned to the Y. pestis genome (NC_003143.1) and plasmids pPCP1 (NC_003132.1) and pMT1 (NC_003134.1) using STAR (60). Read counts for each gene were calculated using htseq-count (61) in conjunction with a gene annotation file for Y. pestis. Read counts for sRNA were obtained using bedtools and the annotated locations reported previously by Schiano et al. (33). Normalization and differential expression were determined using DESeq2 (62). The cutoff for determining significantly differentially expressed genes was a false-discovery-rate (FDR)-adjusted *P* value of less than 0.05. Raw and processed data were deposited into the GEO database at the NCBI (see below).

For validation of RNA sequencing results, qRT-PCR was performed using a SuperScript III Platinum SYBR green one-step qRT-PCR kit (Thermo Fisher Scientific) with 25 ng of DNased RNA on a Bio-Rad iQ5 cycler with melt curve analysis following the manufacturer’s instructions.

### Purification of Y. pestis Crp.

The amplified *crp* gene corresponding to residues 1 to 210 from Y. pestis CO92 (www.csgid.org; accession no. IDP97063) was cloned by Gibson assembly into the SspI site of expression vector pMCSG7 (63, 76), which contains an N-terminal polyhistidine tag followed by the tobacco etch virus (TEV) protease cleavage site and the start codon of the *crp* gene. The resulting plasmid was transformed into E. coli BL21(DE3) (Magic). The bacteria were grown at 37°C and 200 rpm in 3 liters of Terrific broth until the OD_600_ reached 1.6. Protein expression was induced with 0.6 mM IPTG (isopropyl-β-d-thiogalactopyranoside), and cells were grown overnight with the shaking rate reduced to 180 rpm and the temperature to 22°C. Cells were harvested by centrifugation. The resulting cell pellet was resuspended in 120 ml of lysis buffer [10 mM Tris-HCl (pH 8.3), 500 mM NaCl, 1 mM Tris(2-carboxyethyl)phosphine hydrochloride (TCEP), 10% (vol/vol) glycerol, 0.01% (vol/vol) IGEPAL CA630, Roche EDTA-free protease inhibitors (1 tablet/100 ml buffer)], and the suspension was frozen at –20°C until purification. The frozen suspension was thawed under the cold running water, sonicated, and centrifuged. The protein was purified in two steps using nickel (II) affinity chromatography followed by size exclusion chromatography as described previously (65). The polyhistidine tag was removed by incubation of the tagged protein with the recombinant TEV protease for overnight at 20°C. The resulting 58 mg of pure protein was at a final concentration of 13.7 mg/ml.

### Structure determination of Y. pestis Crp.

For crystallization screening, we used a protein solution with a concentration of 6.6 mg/ml in a mixture containing 10 mM Tris-HCl (pH 8.3), 500 mM NaCl, 5 mM TCEP, and 1 mM cAMP. Crystallization drops were equilibrated at a 1:1 protein/reservoir solution ratio against 96 conditions/screen using commercially available PACT, PEG and PEG II suites (Qiagen). Diffraction-quality crystals of Crp were grown under PACT suite condition H12.

Prior to flash-cooling in liquid nitrogen, crystals of Crp were transferred into a 5-μl drop of the reservoir solution that they had grown from. Data were collected on the LS-CAT 21-ID-F beamline at the Advanced Photon Source (APS) at Argonne National Laboratory. A total of 350 images were indexed, scaled, and integrated using HKL-3000 (66). Data collection and data processing statistics are listed in Table S1. The structure of Y. pestis Crp in complex with cAMP was solved by molecular replacement using Phaser (67) from the CCP4 suite (68). The structure E. coli Crp (PDB entry 3RYP) was used as a search model. The initial solution was processed by several rounds of refinement in REFMAC v.5.5, residues were mutated, and cAMP was added in Coot (69). Water molecules were generated using ARP/wARP (70), and the model refinement was continued in REFMAC. Translation-libration-screw (TLS) groups were created by the use of the *TLSMD* server (71) (http://skuld.bmsc.washington.edu/~tlsmd/), and TLS corrections were applied during the final stages of refinement. MolProbity (64; http://molprobity.biochem.duke.edu/) was used for monitoring the quality of the model during refinement and for the final validation of the structure. The final model and diffraction data were deposited in the Protein Data Bank (https://www.rcsb.org/; see below). The final model consists of two polypeptide chains which form a dimer. Chain A contains Crp residues 6 to 208, and chain B contains residues 9 to 208. The crystal structure includes one cAMP molecule bound to each monomer, 3 chloride ions, and 292 water molecules. Refinement statistics and the quality of the final model are summarized in Table S1. Molecular graphics procedures and alignment with E. coli Crp bound to cAMP (PDB ID 4R8H) (30) were performed with the UCSF Chimera package.

10.1128/mBio.02613-19.4 TABLE S1 X-ray data collection and refinement statistics of Y. pestis Crp. Download Table S1, DOCX file, 0.02 MB. Copyright © 2019 Ritzert et al. 2019 Ritzert et al. This content is distributed under the terms of the Creative Commons Attribution 4.0 International license.

### Gel shift assays.

EMSAs were carried out using a LightShift chemiluminescent EMSA kit (Thermo Fisher Scientific). Biotinylated primers were used for PCR amplification of gene-specific promoters (Table S4). PCR products were concentrated and gel purified using Wizard SV Gel and PCR cleanup protocol (Promega). Binding reactions were performed with the manufacturer’s binding buffer, 100 ng poly(dI-dC), and 20 fmol of biotinylated promoter fragment in a 20-μl reaction mixture. Reaction mixtures contained increasing amounts (6.25 to 100 ng) of purified Crp protein and cAMP (Sigma) (2 mmol). Binding reactions were carried out following the manufacturer’s instructions. Nylon membranes (Hybond-N+; 30 cm) were developed using X-ray film.

### GFP reporter assays.

For GFP reporter assays, 2-ml volumes of overnight Y. pestis cultures in TMH medium with 0.2% glucose were subcultured at an OD_620_ of 0.1 into 2 ml of TMH medium incubated at 37°C in a rotary drum for 6 h. Cultures were diluted to an OD_620_ of 0.25 to 0.40, and 200-μl aliquots were added in duplicate to a 96-well plate. Fluorescence was read on a Tecan Safire II plate reader, and results are reported as previously described (6).

### AHL and AI-2 bioreporter assays.

R. radiobacter carrying pZLR4 was grown overnight at 30°C. Cultures were diluted to an OD_620_ of 0.1 in 1 ml of BHI medium. A 1-μl volume of filter-sterilized supernatants of Y. pestis or 10 μl of filter-sterilized BALF, previously collected from mice intranasally infected with wild-type Y. pestis (26), was added. Tubes were incubated at 30°C with shaking at 250 rpm for 5 h. Cultures were pelleted and resuspended in 500 μl of Z-buffer (60 mM Na_2_HPO_4_, 40 mM NaH_2_PO_4_, 10 mM KCl, 1 mM MgSO_4_, pH 7.0). A 25-μl volume of chloroform and a 12.5-μl volume of 0.1% SDS were used to lyse the bacteria for 5 min. A 100-μl volume of 2-nitrophenyl-β-d-galactopyranoside (ONPG; 4 mg/ml) was added to initiate the galactosidase reaction, which was stopped by the addition of 250 μl of 1 M Na_2_CO_3_. The reaction mixtures were centrifuged for 5 min at 13,000 rpm, and 500 μl was added to a cuvette to record the OD_420_. Relative beta-galactosidase activity levels were expressed as ratios of OD_420_/OD_620_.

For TLC, 100-μl volumes of culture supernatants or BALF were extracted twice with 100 μl of ethyl acetate and concentrated to 20 μl in a 60-Hz Savant SpeedVac DNA 100 concentrator (Thermo Fisher Scientific). Volumes of 1 μl of culture supernatants or 7.5 μl of BALF samples were spotted onto aluminum-backed C18-W silica plates (Sorbent Technologies) and developed in 60% methanol–40% water as described previously (55).

To measure AI-2 concentrations, overnight cultures of MM32 were diluted 1:500 and grown at 30°C for 1 h. A 180-μl volume was added to a 96-well plate with 20 μl of either culture supernatant or BALF. Plates were incubated at 30°C with shaking for 5 h. Optical density and luminescence were recorded on a Molecular Devices Spectramax M5 microplate reader. Reported data are from 3 h of incubation (culture supernatants) and 4 h of incubation (BALF).

### Measurement of intracellular cAMP concentration.

Overnight cultures of Y. pestis were diluted to an OD_620_ of 0.1 in TMH medium with 0.2% glucose or 0.2% glycerol and grown for 6 h at 26°C or 37°C. Alternatively Y. pestis was grown in TMH medium overnight in 125-ml Erlenmeyer flasks to form biofilms. Volumes consisting of 0.2 OD_620_ equivalents were centrifuged and lysed in 0.1 M HCl–0.5% Triton X-100 for 10 min. Cellular debris was pelleted by centrifugation, and the supernatant was stored at –20°C. A Direct cAMP ELISA kit (72) was used following the acetylation protocol. cAMP concentrations were normalized to protein content measured using a Pierce bicinchoninic acid (BCA) protein assay kit (Thermo Fisher Scientific).

### Statistical analysis and graphing.

Statistical analyses, including Student’s *t* tests and one-way analysis of variance (ANOVA) with Bonferroni multiple-comparison tests, were performed using GraphPad Prism, version 5.0, with a *P* value of <0.05 as a cutoff for significance. Venn diagrams and volcano plots were generated in R (Version 9.0) using the VennDiagram (Version 1.6.20) and tidyverse (Version 1.1.1) packages.

### Gene ontology analysis.

RNA-seq data were processed at GeneOntology.org (43, 44) using GO enrichment analysis (73). The PANTHER overrepresentation test (released 11 July 2019) was conducted with the GO Ontology database (released 3 July 2019). The reference list used was that of Yersinia pestis.

### Data availability.

Raw and processed data were deposited in the GEO database at the NCBI (GEO accession number GSE135228). The final model and diffraction data for Crp were deposited in the Protein Data Bank (https://www.rcsb.org/) with the assigned PDB entry 6DT4.
