# Supplementary figures and images for: The Cyclic AMP Receptor Protein Regulates Quorum Sensing and Global Gene Expression in Yersinia pestis during Planktonic Growth and Growth in Biofilms
Source: mBio. 2019 Nov 19;10(6):e02613-19. doi: 10.1128/mBio.02613-19 (PMC6867900; doi:10.1128/mBio.02613-19)

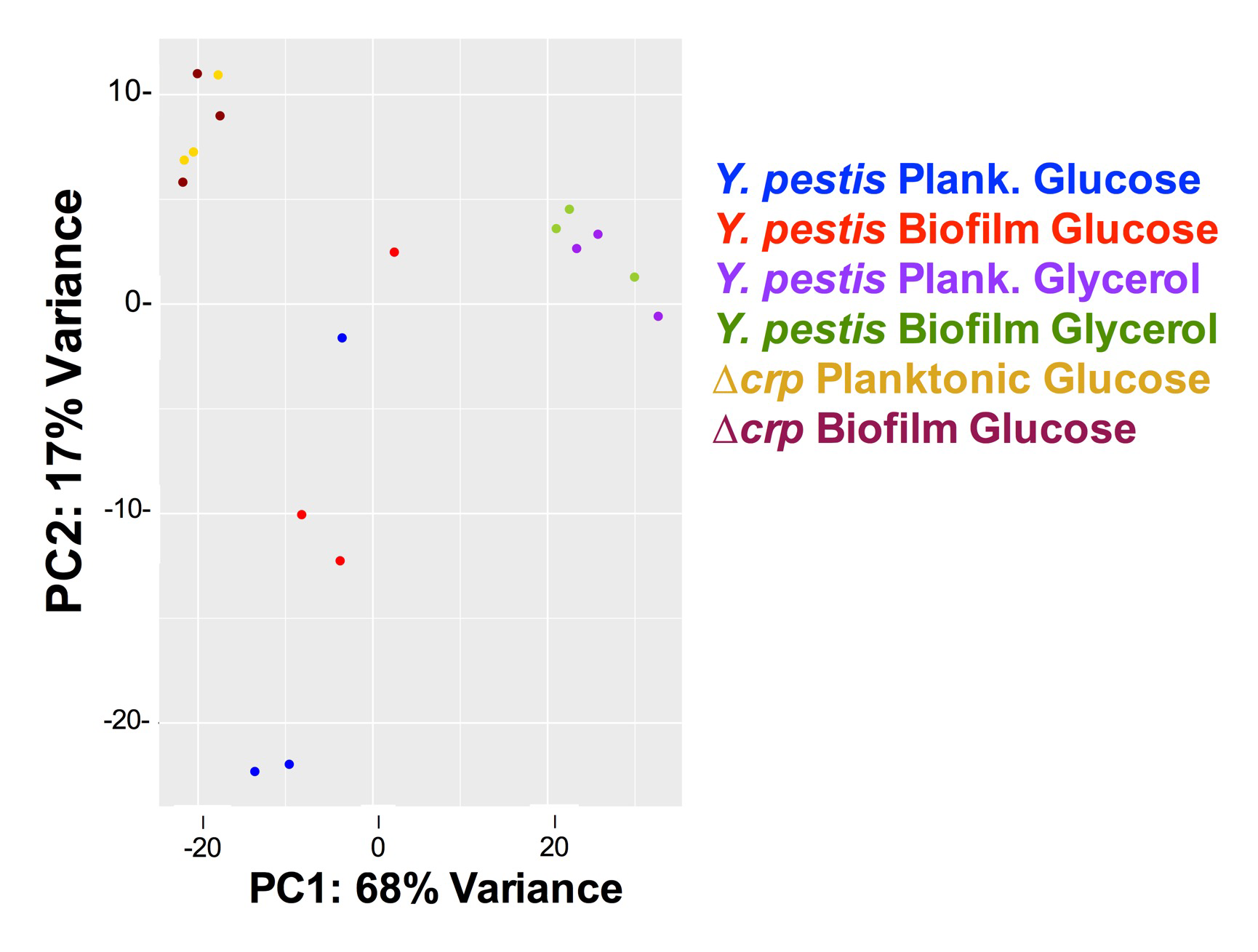

Supplement: FIG S1 [file mBio.02613-19-sf001.tif]

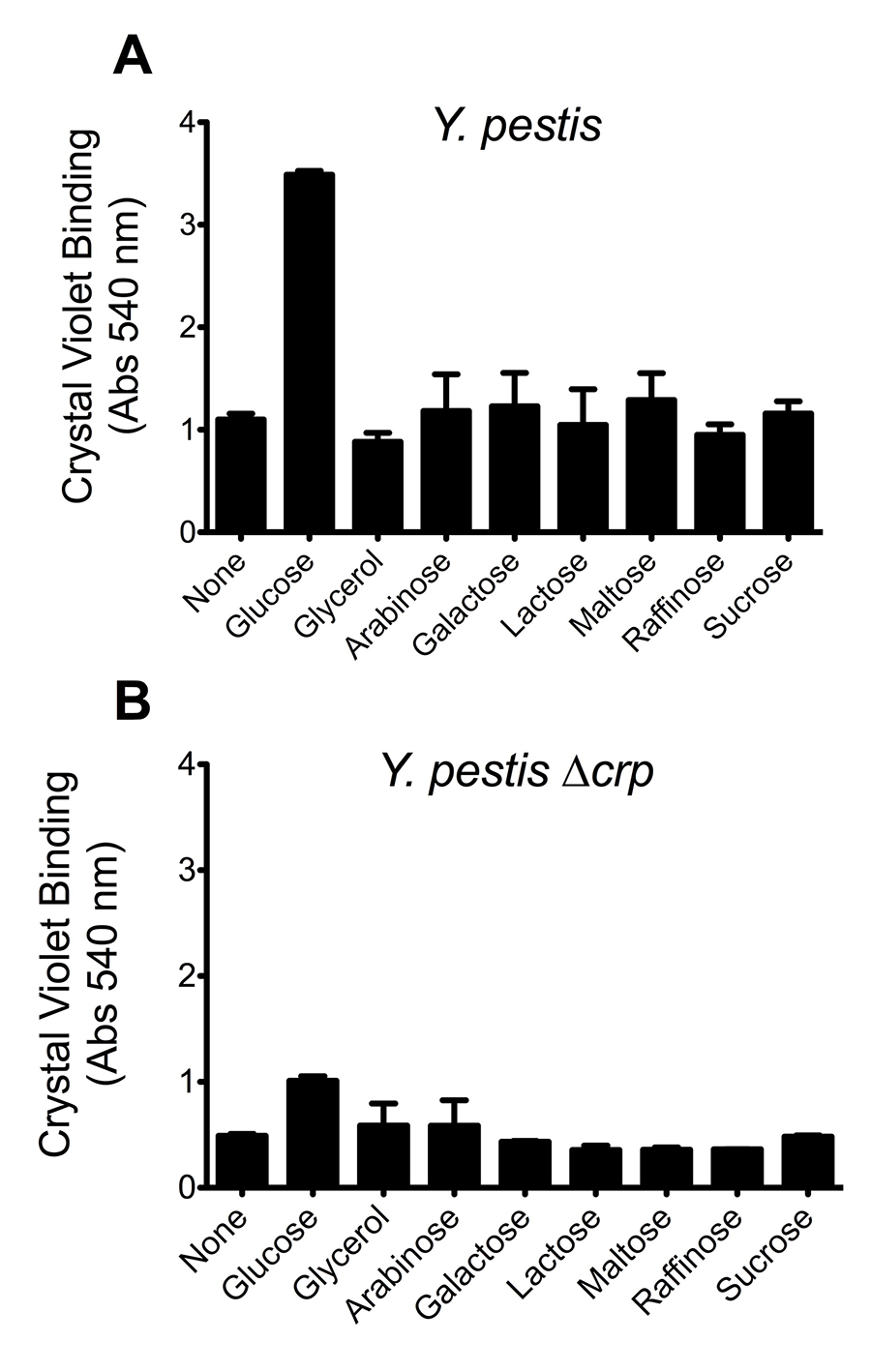

Supplement: FIG S2 [file mBio.02613-19-sf002.tif]

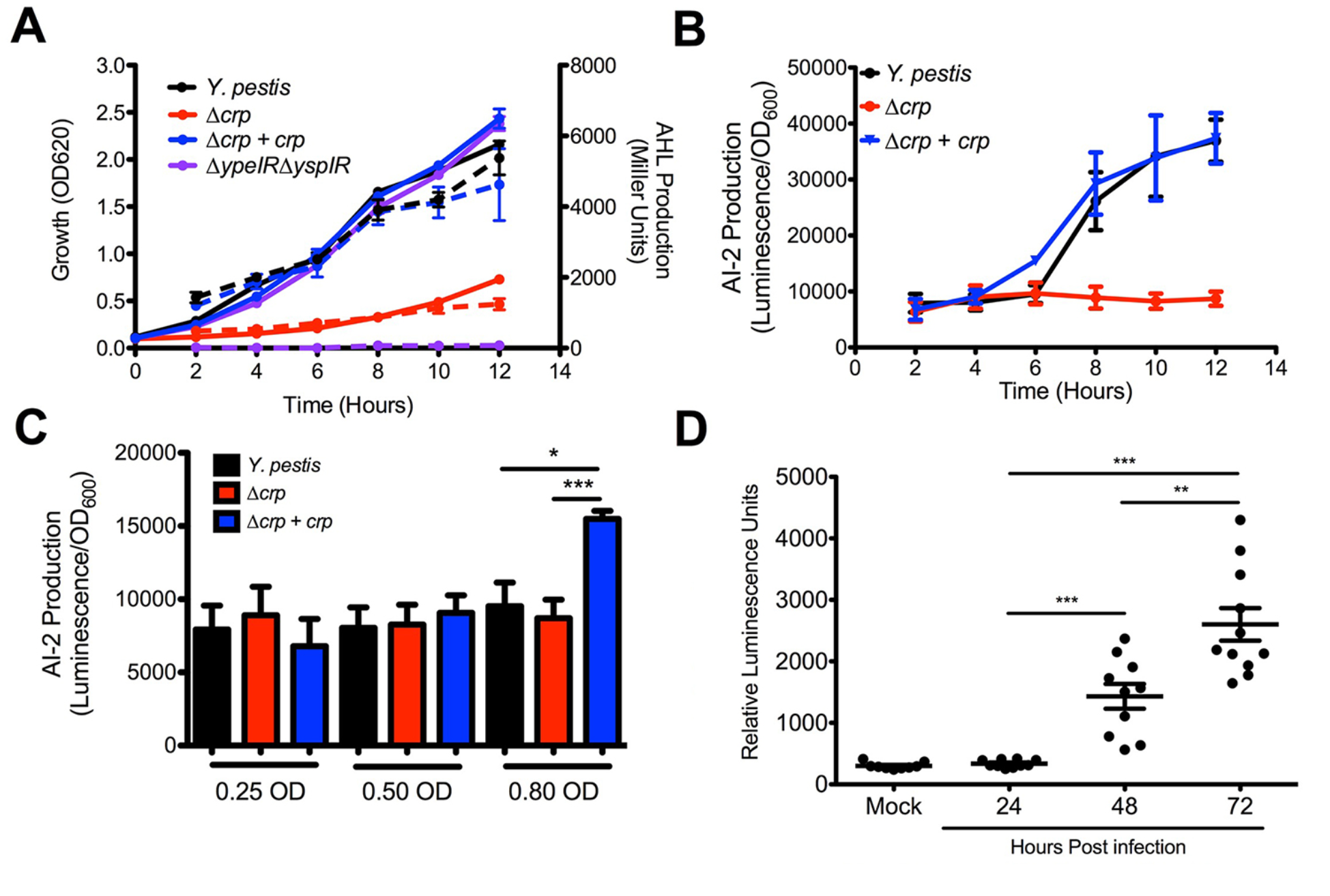

Supplement: FIG S3 [file mBio.02613-19-sf003.tif]
