# Supplementary material for: The Cyclic AMP Receptor Protein Regulates Quorum Sensing and Global Gene Expression in Yersinia pestis during Planktonic Growth and Growth in Biofilms
Source: mBio. 2019 Nov 19;10(6):e02613-19. doi: 10.1128/mBio.02613-19 (PMC6867900; doi:10.1128/mBio.02613-19)
Supplement: TABLE S2 [file mBio.02613-19-st002.docx]

|  | **TABLE S2. Crp-activated and Crp-repressed genes.** | | | | | |
| --- | --- | --- | --- | --- | --- | --- |
|  | Crp-activated^1^ | | | | Crp-repressed^2^ | |
| Planktonic | ***ansB*** | *ypeI* | ***ypo1749*** | ***ypo3006*** | *acpS* | *nuoM* |
|  | *caf1M* | ***ypeR*** | ***ypo1761*** | *ypo3136* | *bioC* | *nuoN* |
|  | ***celA*** | ***YPMT1.70*** | ***ypo1883*** | ***ypo3228*** | *cydC* | ***ptsP*** |
|  | ***crp*** | ***ypo0007*** | ***ypo1886*** | ***ypo3287*** | ***cysA*** | ***purE*** |
|  | *cspE* | *ypo0176* | ***ypo1887*** | *ypo3318* | ***deaD*** | ***purK*** |
|  | *cycA* | ***ypo0277*** | *ypo1904* | ***ypo3472*** | *envZ* | *putA* |
|  | *dadA* | ***ypo0315*** | ***ypo1986*** | ***ypo3514a*** | *fhuD* | *recB* |
|  | ***fadD*** | ***ypo0402*** | ***ypo1989*** | ***ypo3619*** | ***flgH*** | *recD* |
|  | ***fucR*** | *ypo0424* | ***ypo2126*** | ***ypo3647*** | *ftsI* | *recF* |
|  | ***glpF*** | ***ypo0628*** | *ypo2127* | *ypo3648* | *ftsL* | *recJ* |
|  | ***imm*** | ***ypo0749*** | ***ypo2220*** | ***ypo3681*** | *ftsW* | *rnr* |
|  | ***kbl*** | *ypo0760* | *ypo2277* | *ypo3682* | *glgC* | *rpmF* |
|  | ***malT*** | ***ypo0856*** | ***ypo2278*** | ***ypo3874*** | *glgP* | *visC* |
|  | ***mlc*** | ***ypo0867*** | *ypo2279* | *ypo3995* | ***glyS*** | *wrbA* |
|  | ***mtta1*** | ***ypo0884*** | *ypo2337* | *ypo4020* | *hemY* | *ypo0131* |
|  | ***ompC2*** | ***ypo0959*** | ***ypo2444*** | ***ypo4036*** | *iucD* | *ypo0147* |
|  | ***pim*** | ***ypo0987*** | *ypo2472* | ***ypo4041*** | *livG* | *ypo0381* |
|  | ***pla*** | ***ypo1011*** | *ypo2481* | ***YPPCP1.06*** | *mgtE* | *ypo0569a* |
|  | ***psaE*** | ***ypo1174*** | ***ypo2589*** | ***YPPCP1.08c*** | *murC* | *ypo1242* |
|  | ***rbsK*** | ***ypo1235*** | ***ypo2590*** | ***yspI*** | *murD* | *ypo1243* |
|  | ***rcsB*** | ***ypo1255*** | *ypo2729* |  | *murE* | *ypo1981* |
|  | ***rnk*** | ***ypo1382*** | ***ypo2730*** |  | ***murF*** | *ypo2840* |
|  | *sfcA* | ***ypo1454*** | ***ypo2863*** |  | ***murG*** | *ypo3056* |
|  | ***wbyK*** | ***ypo1496*** | ***ypo2955*** |  | *nuoE* | *ypo3435* |
|  | ***ydjJ*** | *ypo1574* | ***ypo2962*** |  | *nuoF* | *ypo3452* |
|  | ***ymoA*** | ***ypo1620*** | ***ypo2980*** |  | *nuoH* | *ypo3453* |
|  | ***yobD*** | ***ypo1718*** | ***ypo3004*** |  | *nuoI* | *ypo3523* |
| Biofilm | *aidB* | *YPMT1.69* | *ypo1234* | ***ypo2863*** | *aceA* | *leuB* |
|  | ***ansB*** | ***YPMT1.70*** | ***ypo1235*** | *ypo2864* | *cmr* | *leuC* |
|  | *argR* | *YPMT1.73* | ***ypo1255*** | *ypo2884* | *cysA* | *luxS* |
|  | ***celA*** | ***ypo0007*** | *ypo1341* | ***ypo2955*** | *cysI* | *menC* |
|  | ***crp*** | *ypo0099* | *ypo1342* | ***ypo2962*** | *cysJ* | *menE* |
|  | ***fadD*** | *ypo0259* | ***ypo1382*** | *ypo2963* | *cysK* | *murF* |
|  | *fadH* | *ypo0276* | ***ypo1454*** | ***ypo2980*** | *cysP* | *murG* |
|  | *fruB* | ***ypo0277*** | *ypo1463* | *ypo3002* | *deaD* | *ntpA* |
|  | ***fucR*** | ***ypo0315*** | *ypo1465* | *ypo3003* | *fabA* | *ompX* |
|  | *glpD* | *ypo0339* | *ypo1474* | ***ypo3004*** | *flgG* | *pdxA* |
|  | ***glpF*** | ***ypo0402*** | *ypo1477a* | ***ypo3006*** | *flgH* | *proW* |
|  | *gutB* | *ypo0523* | *ypo1484* | *ypo3137* | *fyuA* | *ptsP* |
|  | *hexR* | *ypo0536* | *ypo1484a* | *ypo3220* | *glyQ* | *purE* |
|  | *hpaR* | *ypo0598* | *ypo1492* | ***ypo3228*** | *glyS* | *purK* |
|  | *hxuB* | *ypo0601* | ***ypo1496*** | ***ypo3287*** | *guaA* | *purL* |
|  | ***imm*** | ***ypo0628*** | *ypo1498* | *ypo3351* | *guaB* | *recQ* |
|  | *insA* | *ypo0688* | ***ypo1620*** | ***ypo3472*** | *hemF* | *sodC* |
|  | ***kbl*** | *ypo0689* | *ypo1651* | ***ypo3514a*** | *hmuU* | *tolQ* |
|  | *kdgC* | *ypo0690* | *ypo1671* | *ypo3531* | *hscA* | *ybbA* |
|  | ***malT*** | *ypo0691* | *ypo1687a* | *ypo3611a* | *htpG* | *ybiT* |
|  | *manX* | ***ypo0749*** | *ypo1707* | ***ypo3619*** | *ileS* | *ygbE* |
|  | ***mlc*** | *ypo0750* | ***ypo1718*** | ***ypo3647*** | *ilvA* | *yjbA* |
|  | ***mtta1*** | *ypo0759* | *ypo1739* | ***ypo3681*** | *ilvC* | *ypo2036* |
|  | *ogl* | *ypo0764* | ***ypo1749*** | *ypo3683* | *ilvD* | *ypo2622* |
|  | *ogt* | *ypo0768* | ***ypo1761*** | *ypo3709* | *ilvE* | *ypo3033* |
|  | ***ompC2*** | *ypo0804* | *ypo1811* | *ypo3803* | *ilvM* | *ypo3791* |
|  | *pckA* | *ypo0806* | ***ypo1883*** | *ypo3839* |  |  |
|  | ***pim*** | *ypo0808* | ***ypo1886*** | *ypo3840* |  |  |
|  | ***pla*** | *ypo0809* | ***ypo1887*** | ***ypo3874*** |  |  |
|  | ***psaE*** | *ypo0813* | ***ypo1986*** | *ypo3885* |  |  |
|  | *psaF* | *ypo0814* | ***ypo1989*** | *ypo3893* |  |  |
|  | ***rbsK*** | *ypo0833* | *ypo2073* | *ypo3922* |  |  |
|  | ***rcsB*** | ***ypo0856*** | ***ypo2126*** | *ypo3923* |  |  |
|  | *rhaR* | ***ypo0867*** | *ypo2169* | *ypo3935* |  |  |
|  | ***rnk*** | *ypo0874* | ***ypo2220*** | *ypo3953* |  |  |
|  | ***wbyK*** | *ypo0881* | ***ypo2278*** | *ypo3981* |  |  |
|  | *wbyL* | *ypo0882* | *ypo2282* | *ypo3983* |  |  |
|  | *wzz* | *ypo0883* | ***ypo2337*** | *ypo4031* |  |  |
|  | *yapA* | ***ypo0884*** | ***ypo2444*** | ***ypo4036*** |  |  |
|  | *ybcI* | *ypo0947* | *ypo2482* | *ypo4040* |  |  |
|  | ***ydjJ*** | ***ypo0959*** | *ypo2503* | ***ypo4041*** |  |  |
|  | *ydjM* | *ypo0965* | *ypo2531a* | *ypo4049* |  |  |
|  | *yfiA* | *ypo0978* | ***ypo2589*** | *ypo4050* |  |  |
|  | *ylaC* | *ypo0982* | ***ypo2590*** | *ypo4081* |  |  |
|  | ***ymoA*** | ***ypo0987*** | *ypo2591* | ***YPPCP1.06*** |  |  |
|  | ***yobD*** | ***ypo1011*** | *ypo2611* | ***YPPCP1.08c*** |  |  |
|  | ***ypeR*** | *ypo1088* | ***ypo2730*** | *YPPCP1.09c* |  |  |
|  | *YPMT1.53c* | ***ypo1174*** | *ypo2733* | ***yspI*** |  |  |
|  | *YPMT1.68A* | *ypo1233* | *ypo2778* |  |  |  |
|  | ^1^Decreased expression in ∆*crp* vs. *Y. pestis* and glucose vs. glycerol (Log2 FC<1, FDR p<0.05) | | | | | |
|  | ^2^Increased expression in ∆*crp* vs. *Y. pestis* and glucose vs. glycerol (Log2 FC>1, FDR p<0.05) | | | | | |
|  | **Bolded genes** are Crp-activated or Crp-repressed in planktonic and biofilm states. | | | | | |
