# Supplementary material for: The Cyclic AMP Receptor Protein Regulates Quorum Sensing and Global Gene Expression in Yersinia pestis during Planktonic Growth and Growth in Biofilms
Source: mBio. 2019 Nov 19;10(6):e02613-19. doi: 10.1128/mBio.02613-19 (PMC6867900; doi:10.1128/mBio.02613-19)
Supplement: TABLE S3 [file mBio.02613-19-st003.docx]

| **TABLE S3** Bacterial Strains and Plasmids used in this study | | |
| --- | --- | --- |
| Strain or plasmid | Relevant description | Source or Reference |
| ***Yersinia pestis* CO92** |  |  |
| SAN3 | pCD1+, *pgm+,* pMT1+ | (74) |
| PAN30 | pCD1-, ∆*cyaA* | This study |
| PAN259 | pCD1-, “wild-type” | (74) |
| PAN933 | pCD1-, ∆*crp* | (6) |
| PAN977 | pCD1-, ∆*ypeIR* | This study |
| PAN978 | pCD1-, ∆*yspIR* | This study |
| PAN981 | pCD1-, ∆*luxS* | This study |
| PAN985 | pCD1-, ∆*ypeIR*∆*yspIR* | This study |
| PAN1071 | pCD1-, ∆*malT* | This study |
| PAN1072 | pCD1-, ∆*crp* + *crp* | This study |
| PAN1073 | pCD1-, ∆*malT* + *malT* | This study |
|  |  |  |
| ***Rhizobium radiobacter*** |  |  |
| PAN1045 | pZLR4 | ATCC® BAA-2240 |
|  |  |  |
| ***Vibrio harveyi*** |  |  |
| PAN980 | MM32 | (75) |
|  |  |  |
| ***Escherichia coli*** |  |  |
| CC118 λ*pir* | *Δ(ara-leu) araD ΔlacX74 galE galK phoA20 thi-1 rpsE rpoB argE(Am) recAl λpir* | (72) |
| DH5α | *F^-^,*ϕ80d*lacZ*ΔM15 Δ*(lacZYA-argF)*U169 *deoR recA1 endA1 hsdR17(*rK^-^, mK^+^*) phoA supE44 λ*^-^ *thi-1* | Laboratory stock |
| DH5α λ*pir* | As above with λ*pir* | Laboratory stock |
| S17-1 λ*pir* | Tp^R^ Sm^R^ *recA*, *thi-1*, *pro*, *hsdR*^-^ M+RP4: 2-*Tc*:*Mu*: Km Tn*7* λpir | Laboratory stock |
| TOP10 | F^–^ *mcrA Δ(mrr-hsdRMS-mcrBC)* φ80*lacZΔ*M15 *ΔlacX74 recA1 araD139 Δ(ara-leu)7697 galU galK λ^–^ rpsL(Str^R^) endA1 nupG* | Invitrogen |
| BL21(DE3)(Magic) | Overexpression of proteins expressed under T7 promoter with plasmid expressing rare codons, Km^R^ | A. Joachimiak, Argonne National Labs |
|  |  |  |
| **Plasmids** |  |  |
| pWL213 | Constitutive expression of *gfp,* Km^R^ | (35) |
| pUC18 | pUC18R6K-mini-Tn7t, Km^R^ | (58) |
| pTNS2 | Conjugative helper plasmid Amp^R^ | (58) |
| pKD13 | Source of pSkippy Kan cassette Km^R^ | Laboratory Stock |
| pSkippy | IPTG-inducible FLP recombinase, Amp^R^ | Laboratory Stock |
| pWL204 | Lambda red recombinase, Amp^R^ | Laboratory Stock |
| pMCSG53 | Vector for protein overexpression Amp^R^ | (76) |
| pIDP97063 | Overexpression of 6xHis-tagged CRP | This study |
| pLB30 | Promoterless *gfp* for insertion into *Y. pestis* Tn*7* *att* site, Km^R^ | (35) |
| pJR32 | P*ypeI*-*gfp,* Amp^R^ | This study |
| pJR33 | P*yspI*-*gfp,* Amp^R^ | This study |
| pJR34 | P*yspR*-*gfp,* Amp^R^ | This study |
| pJR35 | P*ypeR-gfp,* Amp^R^ | This study |
| pJR36 | *crp* complementation, Amp^R^ | This study |
| pJR37 | *malT* complementation, Amp^R^ | This study |
