# Supplementary material for: The Cyclic AMP Receptor Protein Regulates Quorum Sensing and Global Gene Expression in Yersinia pestis during Planktonic Growth and Growth in Biofilms
Source: mBio. 2019 Nov 19;10(6):e02613-19. doi: 10.1128/mBio.02613-19 (PMC6867900; doi:10.1128/mBio.02613-19)
Supplement: TABLE S4 [file mBio.02613-19-st004.docx]

| **TABLE S4** Oligonucleotides used in this study | |  |
| --- | --- | --- |
| Designation | Sequence (5’ to 3’) | Purpose |
| 5’ p1 for pKD13 | GTGTAGGCTGGAGCTGCTTC | Amplification Kan cassette for lambda red |
| 3’ p4 for pKD13 | ATTCCGGGGATCCGTCGACC | Amplification Kan cassette for lambda red |
| 5’ -500 malt | TGCTCGGTGCGGGCTGAGCAG | Lambda red deletion of *malt* |
| 3’ +1 malT P1 | GAAGCAGCTCCAGCCTACACTCTGTGTCCGCTGGAAAAG | Lambda red deletion of *malt* |
| 5’ malt p4 | GGTCGACGGATCCCCGGAATCGGGCGCACACGTGTTTGATG | Lambda red deletion of *malt* |
| 3’ +500 malt | TGCATGACCGGCTTCGTAAC | Lambda red deletion of *malt* |
| 5’ -500 ypeIR | CACTCCCCCAAAGTAGACAAC | Lambda red deletion of *ypeIR* |
| 3' P1 ypeIR | GAAGAGCTCCAGCCTACACATTTATTTAATTAAACCAATATC | Lambda red deletion of *ypeIR* |
| 5' P4 ypeIR | GGTCGACGGATCCCCGGAATAGCAGACCAAATTTACTTTATCC | Lambda red deletion of *ypeIR* |
| 3' +500 ypeIR | CGGCTGTGCTTTAGCAGCCC | Lambda red deletion of *ypeIR* |
| 5' -500 yspIR | TCCTCCAGCGCTTTGAGACAC | Lambda red deletion of *yspIR* |
| 3' P1 yspIR | GAAGCAGCTCCAGCCTACACTATCTTTCCTGATATTTAATAC | Lambda red deletion of *yspIR* |
| 5' P4 yspIR | GGTCGACGGATCCCCGGAATTCCCTTTCTCCATTTACTGTAC | Lambda red deletion of *yspIR* |
| 3’ +500 yspIR | AGAATTTATGCGATTCGTGGC | Lambda red deletion of *yspIR* |
| 5’ SpeI -500 crp | TTCGATCATGCATGAGCTCAAAAATAGACACGACATCAATG | Tn7 complementation of *crp* |
| 3' SpeI +500 crp | CCTGCAGCCCGGGGGATCCACCATGCTGAGACTGAAAATAG | Tn7 complementation of *crp* |
| 5’ SpeI -500 malT | TTCGATCATGCATGAGCTCATGCTCGGTGCGGGCTGAGCAG | Tn7 complementation of malt |
| 3’ SpeI +500 malt | CCTGCAGCCCGGGGGATCCATGCATGACCGGCTTCGTAAC | Tn7 complementation of malt |
| 5' -300 pla BT | 5BIOSG/TCTCGCCCGTAAATACCTGAG | Biotinylated primer for amplifying *pla* promoter |
| 3' +1 pla BT | 5BIOSG/TAGACACCCTTAATCTCTCTG | Biotinylated primer for amplifying *pla* promoter |
| 5’ -300 ptsG BT | /5Biosg/TTA GCT CGT AAT TAA TCA CCG | Biotinylated primer for amplifying *ptsG* promoter |
| 3' +1 ptsG BT | /5Biosg/ATA GTT GAG CGT GCT CCT GAG | Biotinylated primer for amplifying *ptsG* promoter |
| 5' -300 malT BT | 5BIOSG/GTGAATGGGTAATTTTACCCG | Biotinylated primer for amplifying *malT* promoter |
| 3' +1 malT BT | 5BIOSG/ATTCTGTGTCCGCTGGAAAAG | Biotinylated primer for amplifying *malT* promoter |
| 5' -300 ypeR BT | 5BIOSG/ATATATTCACAGCAGACTAAC | Biotinylated primer for amplifying *ypeR* promoter |
| 3' +1 ypeR BT | 5BIOSG/AGCAGACCAAATTTACTTTATC | Biotinylated primer for amplifying *ypeR* promoter |
| 5' -300 yspI BT | /5BIOSG/CATGCTACGAGAGAATTATAC | Biotinylated primer for amplifying *yspI* promoter |
| 3' +1 yspI BT | 5BIOSG/TATCTTTCCTGATATTTAATAC | Biotinylated primer for amplifying *yspI* promoter |
| 5’ -300 yspR BT | /5Biosg/GAG GGA CAA TTA ATC ACA ATG | Biotinylated primer for amplifying *yspR* promoter |
| 3' +1 yspR BT | /5Biosg/TCC CTT TCT CCA TTT ACT GTA C | Biotinylated primer for amplifying *yspR* promoter |
| 5' -300 ypeI BT | /5Biosg/CTA AAT ACC ACT CAC ACA AGC | Biotinylated primer for amplifying *ypeI* promoter |
| 3' +1 ypeI BT | /5Biosg/ATT TAT TTA ATT AAA CCA ATA TC | Biotinylated primer for amplifying *ypeI* promoter |
| gyrB.r.3' | ATTGGTAAAGGTCTGGAAACTTGGCC | qRT-PCR of *gyrB* |
| gyrB.f.5' | TCGCCGTGAAGGTAAAGTTC | qRT-PCR of *gyrB* |
| 5’ pla 397 | GACCTCAATGTGAAAGGCTGGTTACACC | qRT-PCR of *pla* |
| 3’ pla 498 | ACCACCTGTAGCTGTCCAACTGAAAC | qRT-PCR of *pla* |
| 5’ ptsG 132 | CTC TCA CGT AAT GGC AGA AG | qRT-PCR of *ptsG* |
| 3’ ptsG 229 | ACC GTC GTT GTT GGT AAA G | qRT-PCR of *ptsG* |
| 5' malt1078 | GCTATCCACCACGCTTTAG | qRT-PCR of *malT* |
| 3' malt 1184 | GTTCCAACAACGCCAATTC | qRT-PCR of *malT* |
| 5’ ybta 282 | TCGCCATTACATTACCCATAC | qRT-PCR of *ybtA* |
| 3’ ybta 414 | CAGTGGGAGTCGATCTTATTC | qRT-PCR of *ybtA* |
| 5’ fyua 848 | AGACCCTGAGTGGGAAATAC | qRT-PCR of *fyuA* |
| 3’ fyua 1027 | GTACAGCCCAAACACCATATC | qRT-PCR of *fyuA* |
| 5’ yspI 27 | CGACGAGTTGACCGATATAC | qRT-PCR of *yspI* |
| 3’ yspI 187 | ACGCACACTGCAGATTAG | qRT-PCR of *yspI* |
| 5’ yper 281 | ATTCGGCCGTGTTCAATC | qRT-PCR of *ypeR* |
| 3’ yper 412 | TGTTCACCTCGATGCTTTC | qRT-PCR of *ypeR* |
| 5’ crp 2 | TGGTTCTCGGTAAGCCACAAACAGAC | qRT-PCR of *crp* |
| 3’ crp 146 | GCAACGGAGCCTTTCACGATGTAG | qRT-PCR of *crp* |
| 5' gfp +1 | ATGAGTAAAGGAGAAGAACTTTTC | Amplification of *gfp* CDS |
| 3' gfp SpeI puc18 | CAGCCCGGGGGATCCACTAGTTATTTGTATAGTTCATCCATG | Amplification of *gfp* CDS for Tn7 |
| 5’ puc18 pypei | TCATGCATGAGCTCACTAGCACTCCCCCAAAGTAGACAAC | Amplification of P*ypeI*-GFP reporter |
| 3’ gfp pypei | AGTTCTTCTCCTTTACTCATAAATACTTTTAACATAATAAAAAC | Amplification of P*ypeI*-GFP reporter |
| 5’ puc18 pyper | TCATGCATGAGCTCACTAGCGGCTGTGCTTTAGCAGCCC | Amplification of P*ypeR*-GFP reporter |
| 3’ gfp pyper | AGTTCTTCTCCTTTACTCATTTCATTATCAAAAAAATTAATTATC | Amplification of P*ypeR*-GFP reporter |
| 5’ puc18 pyspi | TCATGCATGAGCTCACTAGTCCTCCAGCGCTTTGAGACAC | Amplification of P*yspI*-GFP reporter |
| 3’ gfp pyspi | AGTTCTTCTCCTTTACTCATGTATCTGACATCGAAAATTTC | Amplification of P*yspI*-GFP reporter |
| 5’ puc18 pyspr | TCATGCATGAGCTCACTAGGGCTGTAAGAATTTATGCGATTC | Amplification of P*yspR*-GFP reporter |
| 3’ gfp pyspr | AGTTCTTCTCCTTTACTCATGTTACTCCTATTGAAAACAGAATG | Amplification of P*yspR*-GFP reporter |
