# Supplementary material for: The Cyclic AMP Receptor Protein Regulates Quorum Sensing and Global Gene Expression in Yersinia pestis during Planktonic Growth and Growth in Biofilms
Source: mBio. 2019 Nov 19;10(6):e02613-19. doi: 10.1128/mBio.02613-19 (PMC6867900; doi:10.1128/mBio.02613-19)
Supplement: TABLE S1 [file mBio.02613-19-st001.docx]

| **TABLE S1** X-Ray Data Collection and Refinement Statistics of *Y. pestis* Crp | |
| --- | --- |
| Crystal | *Y. pestis* Crp (PDB ID 6DT4) |
| **Data Collection** |  |
| Diffraction Source | Beamline 21ID-F, APS |
| X-ray Wavelength (Å) | 0.97872 |
| Space group) | P 21 21 21 |
| a, b, c (Å) | 53.876, 82.800, 106.350 |
| α, β, γ (°) | 90.00, 90.00, 90.00 |
| Resolution range (Å) | 30.00 – 1.80 (1.83 – 1.80) |
| No. of unique reflections | 44,393 (2,134) |
| Data Completeness (%) | 99.1 (97.3) |
| Multiplicity | 8.5 (7.5) |
| 〈I/σ(I)〉 | 30.5 (2.9) |
| R_sym_ (%) [^†^](https://www.ncbi.nlm.nih.gov/pmc/articles/PMC5683032/table/table3/?report=objectonly#tfn1) | 5.8 (72.2) |
| Wilson plot B factor (Å^2^) | 29.9 |
| **Structure Refinement** |  |
| Resolution range (Å) | 29.61-1.80 (1.85-1.80) |
| Completeness (%) | 99.0 (96.6) |
| No. of observed reflections | 44349 (3122) |
| No. of R _free_ reflections | 2173 (145) |
| Final R _work_ (%) | 17.5 (29.1) |
| Final R _free_ (%) | 20.3 (29.5) |
| **No. of non-H atoms** |  |
| Protein | 3347 |
| Ligand | 49 |
| Water | 312 |
| Total | 3708 |
| **R.m.s. deviations** |  |
| Bonds (Å) | 0.008 |
| Angles (°) | 1.3 |
| **Average B factors** (Å^2^) |  |
| Protein | 42.7 |
| Ligand | 26.6 |
| Water | 44.1 |
| **Ramachandran plot**[^†^](https://www.ncbi.nlm.nih.gov/pmc/articles/PMC5683032/table/table4/?report=objectonly#tfn2) |  |
| Favored regions (%) | 99.0 |
| Additionally allowed (%) | 1.00 |
| Outliers (%) | 0.00 |

[^a^](http://www.cell.com/molecular-cell/fulltext/S1097-2765(15)00902-8#back-tblfn1) Values in parenthesis are for the highest resolution shell.

[^b^](http://www.cell.com/molecular-cell/fulltext/S1097-2765(15)00902-8#back-tblfn2) Rsym = Σ^|^*^I^*^-^*^(I)^*^|^/ Σ^I^ where *I* is the observed intensity of a reflection and *(I)*is the average intensity of all the symmetry related reflections.

[^c^](http://www.cell.com/molecular-cell/fulltext/S1097-2765(15)00902-8#back-tblfn3) For R_free_ calculation, 5% randomly selected reflections were excluded from the refinement.
